# Activating mutations in *ESR1* contribute to an immunosuppressive breast tumor microenvironment by dampening cytokine secretion

**DOI:** 10.1172/jci.insight.199927

**Published:** 2026-03-09

**Authors:** Yu Gu, Dongmei Zuo, Qi-Xin Hu, Virginie Sanguin-Gendreau, Alain Pacis, Marie-Christine Guiot, Alexander Chih-Chieh Chang, Tarek Taifour, Chen Ling, Adrian V. Lee, Steffi Oesterreich, William J. Muller

**Affiliations:** 1 Rosalind and Morris Goodman Cancer Institute and; 2 Faculty of Medicine and Health Sciences, Department of Biochemistry, McGill University, Montreal, Quebec, Canada.; 3 Renmin Hospital of Wuhan University, Wuhan University, Wuhan, China.; 4 Canadian Centre for Computational Genomics, McGill University Genome Center, Montreal, Quebec, Canada.; 5 McGill University Health Centre, Montreal, Quebec, Canada.; 6 Department of Pharmacology and Chemical Biology, University of Pittsburgh, Pittsburgh, Pennsylvania, USA.; 7 Women’s Cancer Research Center, Magee-Womens Research Institute, UPMC Hillman Cancer Center, Pittsburgh, Pennsylvania, USA.; 8 Faculty of Medicine and Health Sciences, Division of Experimental Medicine, and; 9 Faculty of Medicine, McGill University, Montreal, Quebec, Canada.

**Keywords:** Immunology, Oncology, Breast cancer, Cytokines, Mouse models

## Abstract

Patients with estrogen receptor^+^ (ER^+^, *ESR1^+^*) breast cancer are most at risk of relapse, where activating mutations in *ESR1* promote metastasis and therapeutic resistance. These patients are also disadvantaged in responding to immunotherapies, the mechanisms of which remain to be elucidated. Here, we engineered a transgenic mouse model carrying either Y541S or D542G mutation in *ESR1*, mirroring the 2 most common mutations seen in patients. *ESR1^mut^* tumors do not differ in the total number of immune cells yet display downregulation in immune pathways and decreased immune-modulatory cytokines, including IL-17a and IL-1β. T cells and macrophages have lower IFN-γ and antigen presentation, respectively. Mechanistically, *ESR1^mut^* negatively regulates immune modulator expression and upregulates Stat5 to dampen cytokine expression. In concordance, validation on *ESR1^mut^* patient tumors shows decreased IL-17a and IL-1β. Collectively, our findings reveal that *ESR1* mutations contribute to an immunosuppressive tumor microenvironment by dampening cytokine secretion and immune cell activity.

## Abstract 2

Breast tumors with *ESR1* mutations release fewer immune‑boosting cytokines, creating a tumor microenvironment where immune cells have limited ability to eliminate cancer cells.

## Graphical abstract

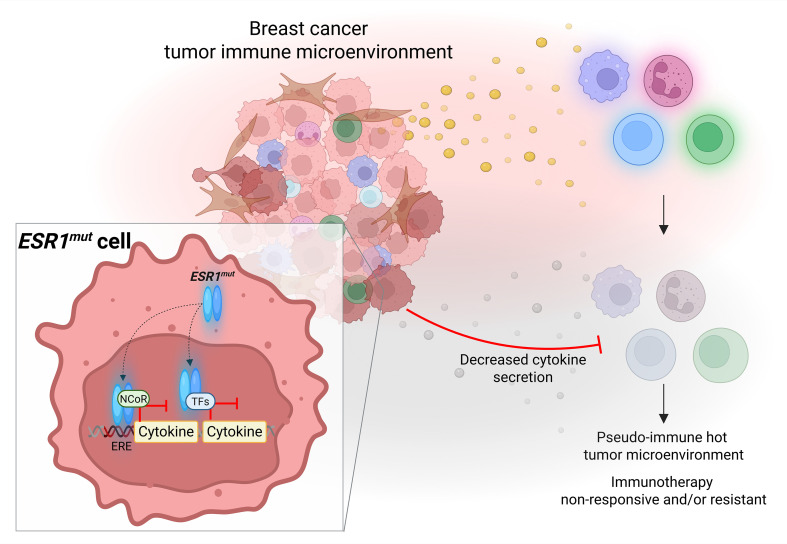

## Introduction

Estrogen-mediated signaling has long been recognized to play a predominant role in the growth and development of breast cancer. The biological activity of estrogens is mediated by 2 estrogen receptors (ERs), both members of the nuclear receptor superfamily (1, 2). ERα (*ESR1*) is expressed in nearly 80% of all breast cancers and is a major regulator of breast cancer development and progression (3–5). Current therapeutic strategies to target ER^+^ breast cancers are either by ligand deprivation (aromatase inhibitors) or ER blockade through selective ER modulators and degraders (6–8). Although these therapies improve survival in early-stage ER^+^ breast cancers, a large proportion of patients develop resistance and late lethal recurrent disease (9–12).

Among the numerous molecular and cellular adaptations in ER^+^ breast tumors, somatic *ESR1* mutations are well-documented mechanisms of endocrine therapy resistance, especially in late-stage metastatic tumors, where 30% of patients present with at least 1 point mutation in *ESR1* (13–15). Clinically, the 2 most frequent constitutively active *ESR1* mutations are tyrosine 537 to serine (*ESR1^Y537S^* in humans, *ESR1^Y541S^* in mice) and aspartic acid 538 to glycine (*ESR1^D538G^* in humans, *ESR1^D542G^* in mice) (16). These mutations in *ESR1*’s ligand-binding domain allow ER to stay in a constitutively active conformation and confer ligand-independent activity (17, 18). Additionally, ER^+^ patients, including *ESR1^mut^* metastatic patients, respond poorly to immunotherapies. However, only a few known mechanisms delineate the *ESR1^mut^* tumor cell–intrinsic and environmental modulations that limit their responsiveness to immunotherapies (19–22).

The paucity of studies on the biological and tumor environmental effects of *ESR1* mutations in primary and metastatic mammary tumorigenesis stems from several limitations, including the lack of in vivo models. In most transgenic mouse models of mammary tumorigenesis, oncogene expression is driven by a hormone-responsive promoter like Whey Acidic Protein (WAP) or Mouse Mammary Tumor Virus (MMTV), which can be influenced by a lack of functional ER (23–27). Here, to circumvent the complications of a hormone-regulated promoter, we introduced an inducible *ESR1^Y541S^* or *ESR1^D542G^* knock-in mutation into the polyoma middle T antigen–driven (PyV mT–driven) transgenic mice, as we have previously shown that *ESR1^mut^* alone is insufficient to drive mammary tumorigenesis (28). Upon doxycycline induction, mammary epithelial cells activate the coupled expression of PyV mT and Cre recombinase for the excision of WT *ESR1* and put in frame the expression of either *ESR1^Y541S^* or *ESR1^D542G^* mutation (MIC *ESR1^mut^* model). Consistent with their clinical manifestations, *ESR1^Y541S/D542G^* mice minimally affect primary mammary tumor kinetics, though tumors demonstrate activation in the ER pathway, with MIC *ESR1^Y541S^* mice developing larger lung metastases. Although no significant differences were observed in the total number of immune cell populations in MIC *ESR1^Y541S^* tumors, they expressed lower levels of activation markers IFN-γ and MHC II for T cells and macrophages, respectively, with decreased immune cytokines, including IL-17a and IL-1β. Further mechanistic investigations revealed that, unlike WT *ESR1*, *ESR1^Y541S^* negatively regulates the expression of immune modulators and cytokine targets. Additionally, *ESR1^Y541S^* tumors upregulate Stat5, known to interact with ER and to transcriptionally repress IL-17a and other immune cytokines. In patients, *ESR1^Y537S^* tumors also exhibit lower levels of IL-17a and IL-1β without notable changes in the levels of total T cells and macrophages, validating our observations in the clinical setting. Collectively, these data support that activating mutations in *ESR1* mediate a repressive mechanism on immunomodulatory cytokines as a transcription factor alone and with Stat5, thus promoting an immunosuppressive tumor microenvironment with dampened T cell and macrophage activities.

## Results

### ESR1^Y541S^ and ESR1^D542G^ mutations increase lung metastatic lesions and activate ER signaling in a PyV mT–driven in vivo model of luminal breast cancer.

**Figure 1:**
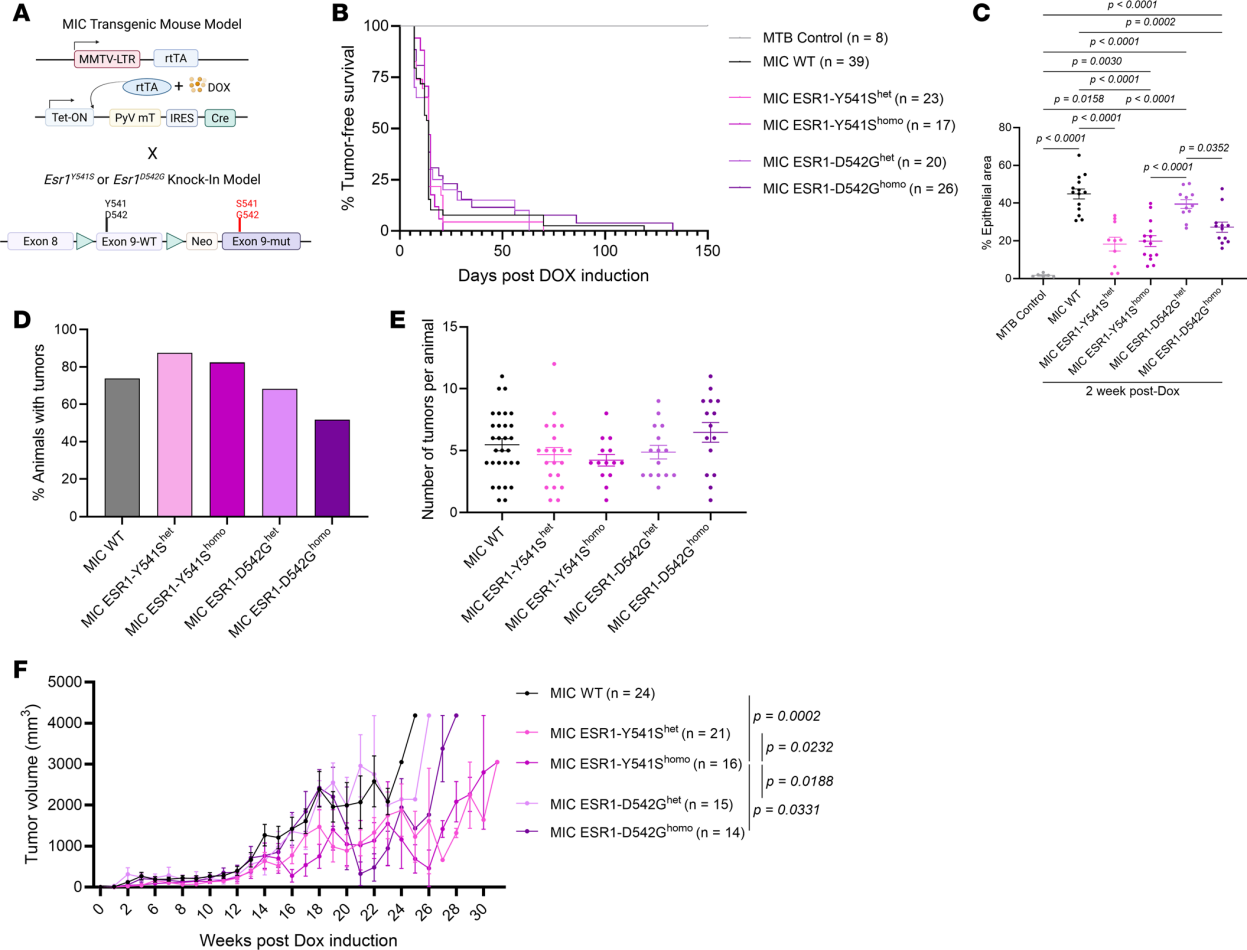
Tumor kinetic characterization of Y541S^het/homo^ and D542G^het/homo^ point mutations in PyV mT–driven mammary tumors. (**A**) Schematic representation of the MIC construct with knocked-in Y541S or D542G point mutation on the *ESR1* gene specifically in the mammary epithelial cells with tumorigenesis driven by the PyV mT antigen. Created in BioRender. (**B**) Percent of tumor-free survival between. Statistical significance was calculated using the log-rank (Mantel-Cox) test. (**C**) Quantification of H&E images of mammary gland (AMG) epithelial transformation area (tumor initiation) beyond 2 weeks after DOX induction (Supplemental Figure 1A). (**D**) Percentage of tumor-bearing animals (tumor penetrance). (**E**) Number of mammary tumors per animal at experimental endpoint. (**F**) Tumor volume measured from weekly palpations. Statistical significance was calculated using the CGGC permutation test. Mean ± SEM for data calculated using 1-way ANOVA with Tukey’s multiple-comparison test unless otherwise indicated.

To investigate the effect of *ESR1* activating point mutations in aggressive luminal breast cancers, *ESR1^Y541S^* or *ESR1^D542G^* were knocked into the previously described doxycycline inducible PyV mT antigen-driven MIC transgenic mouse model (Figure 1A) (26). Cohorts for heterozygous and homozygous knock-in *ESR1* mutant–bearing mice were generated. However, neither mutants nor their homozygosity affected mammary tumor onset (Figure 1B). At the early tumor initiation stage, 2 weeks after doxycycline induction, MIC *ESR1^Y541S^* mammary glands had a noticeable decrease in malignant transformation and hyperplasia, accompanied by a decrease in ER activity readout cyclin D1 levels (Figure 1C and Supplemental Figure 1, A–D; supplemental material available online with this article; https://doi.org/10.1172/jci.insight.199927DS1). In contrast, MIC *ESR1^D542G^* mammary glands displayed comparable transformation to MIC WT mammary glands, and with relatively comparable levels of ER coactivator cyclin D1 and FOXA1 (Figure 1C and Supplemental Figure 1, A–D) (29–32).

At primary mammary tumor endpoint, MIC *ESR1^Y541S^* and *ESR1^D542G^* tumor penetrance and multifocality were comparable with MIC WT mice (Figure 1, D and E). MIC *ESR1^Y541S^* tumor growth rate was delayed compared with MIC WT and to MIC *ESR1^D542G^* cohorts (Figure 1F). MIC WT, *ESR1^Y541S^,* or *ESR1^D542G^* endpoint mammary tumors had comparable pathology with epithelial-dense and distinct stroma areas, characteristic of PyV mT–driven mammary tumors (Supplemental Figure 2A). Although all cohorts exhibited lung metastases at primary tumor endpoint, MIC *ESR1^Y541S^* mice had larger metastatic lesions, reflective of the clinical implication of this mutation in metastatic ER^+^ breast cancers (Supplemental Figure 2, B and C) (33, 34). No mice developed liver metastases, as expected for the MIC model (Supplemental Figure 2D). Lung metastatic lesions from all cohorts were validated to be ER^+^ PyV mT^+^ (Supplemental Figure 2E).

**Figure 2:**
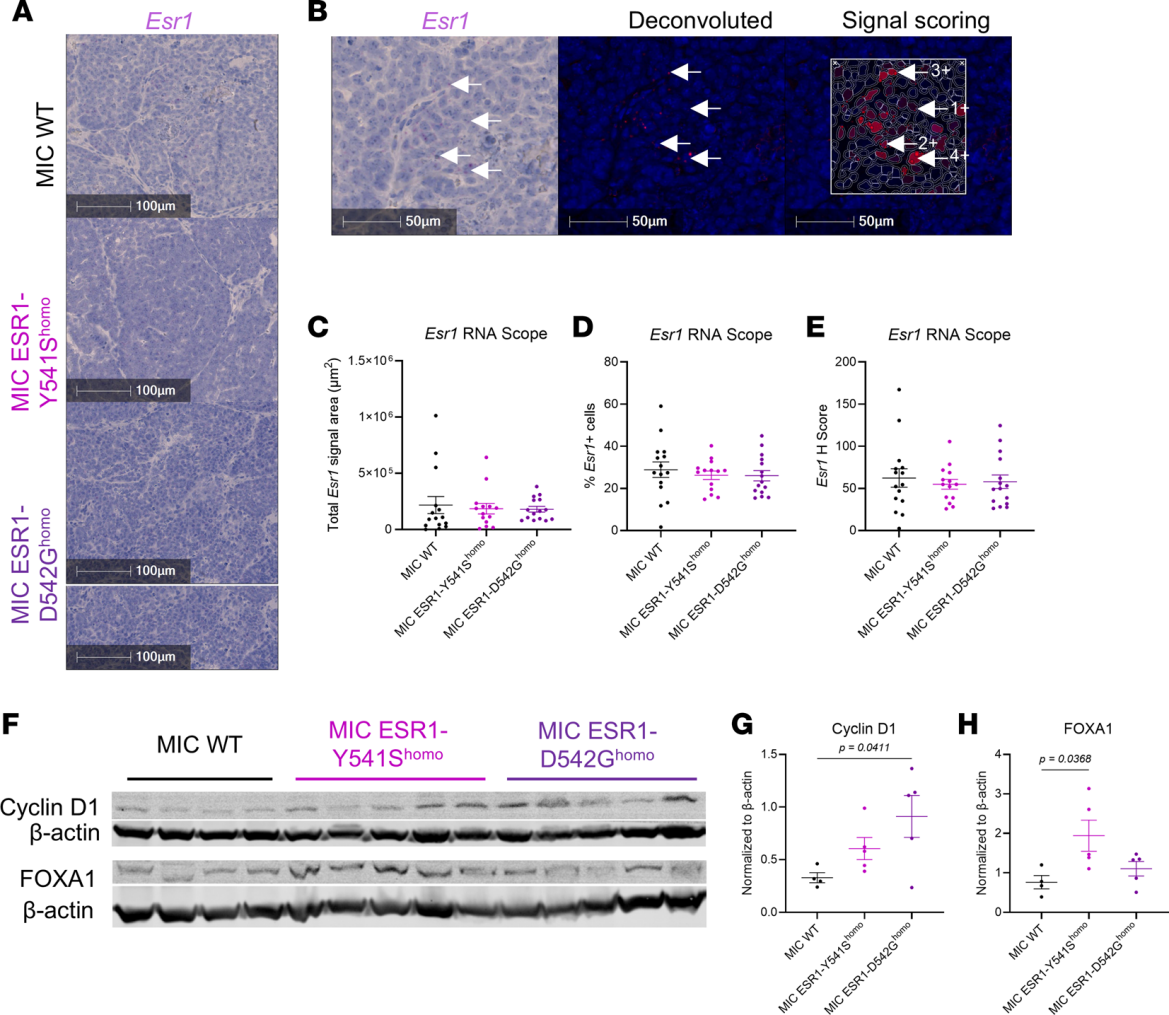
*Esr1* RNA abundance and ER activity validation in MIC WT and ESR1^mut^ tumors. (**A**) RNA fluorescence in situ hybridization (FISH) for mouse *Esr1* and hematoxylin on endpoint mammary tumors of MIC WT, MIC ESR1-Y541S^homo^, and MIC ESR1-D542G^homo^ mice. (**B**) Example of image deconvolution and signal scoring scheme for RNA FISH of mouse *Esr1* based on signal intensity. (**C**–**E**) Quantification of total *Esr1* RNA FISH signal area, *Esr1*^+^ cells, and *Esr1* H score, respectively. (**F**) Immunoblot for cyclin D1 and FOXA1 with their respective loading control (β-actin) on endpoint mammary tumor lysates of MIC WT, MIC ESR1-Y541S^homo^, and MIC ESR1-D542G^homo^ mice. (**G** and **H**) Quantification of immunoblot for cyclin D1 and FOXA1 normalized to their respective loading control, respectively. Scale bars: 100 μm (**A**), 50 μm (**B**). Arrows indicate positive Esr1 RNA-FISH signals. Mean ± SEM for data calculated using 1-way ANOVA with Tukey’s multiple-comparison test.

We then confirmed *Esr1* expression by RNA fluorescence in situ hybridization (FISH), the levels of which did not differ between MIC WT, *ESR1^Y541S^*, or *ESR1^D542G^* tumors for signal area, positive cells, and intensity, with approximately 30% *Esr1* positivity across genotypes (Figure 2, A–E). To validate that the knock-in mutations are activating the ER signaling pathways, MIC *ESR1^Y541S^* and *ESR1^D542G^* tumors indeed displayed increased cyclin D1 and FOXA1 levels (Figure 2, F–H). Collectively, the results support that the active ER signaling and activity observed in MIC *ESR1^mut^* tumors are attributed to the activating mutations and not due to increased *Esr1* transcript number.

### ESR1^Y541S^ and ESR1^D542G^ mutant tumors display downregulation in immune-modulatory pathways with decreased immune cytokine secretion and immune cell activity.

**Figure 3:**
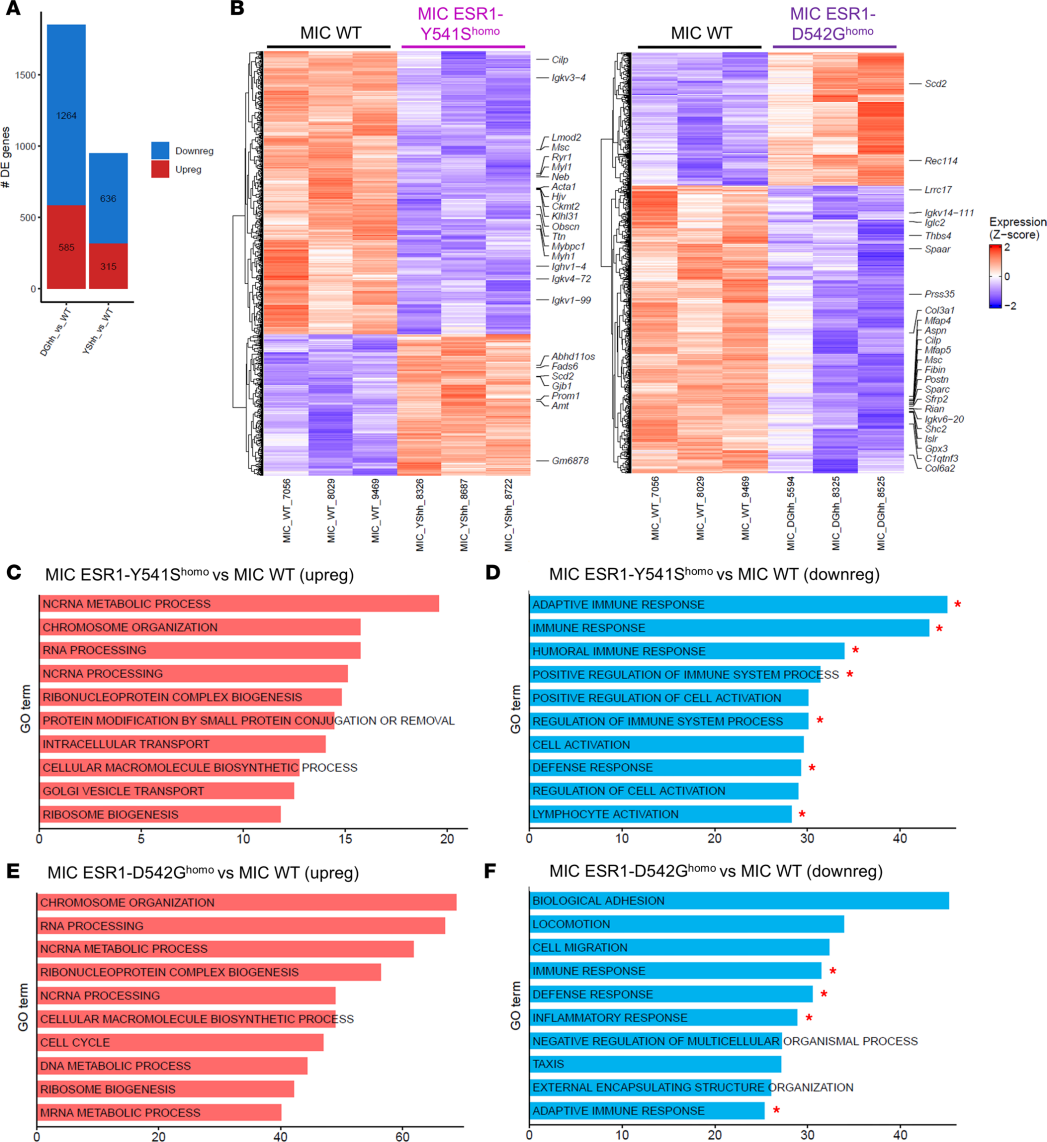
Bulk RNA-seq analyses on MIC WT, MIC ESR1-Y541S^homo^, and MIC ESR1-D542G^homo^ endpoint mammary tumors. (**A**) Number of differentially expressed genes (DEG) between MIC WT and MIC ESR1-Y541S^homo^ or MIC ESR1-D542G^homo^ tumors (*n* = 3 for each group, *P*_adj_ < 0.1). (**B**) Heatmap representation of DEG between MIC WT and MIC ESR1-Y541S^homo^ or MIC ESR1-D542G^homo^ tumors, respectively. (**C** and **D**) Top Gene Ontology (GO) terms identified from upregulated (upreg) and downregulated (downreg) DEG between MIC WT and MIC ESR1-Y541S^homo^ tumors, respectively, with asterisks delineating immune-related pathways. (**E** and **F**) Top GO terms identified from upregulated and downregulated DEG between MIC WT and MIC ESR1-D542G^homo^ tumors, respectively, with stars delineating immune-related pathways. The *x* axes for **C**–**F** are –log_10_(*P*_adj_) as per GO enrichment analysis.

**Figure 4:**
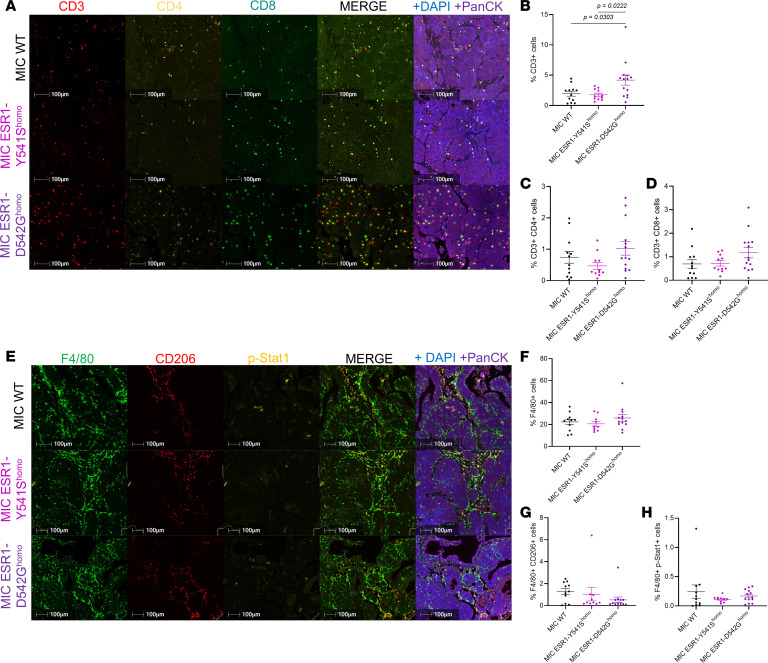
Characterization of T cell and macrophage populations in endpoint mammary tumors. (**A**) Fluorescent IHC for CD3, CD4, CD8, PanCK, and DAPI on endpoint mammary tumors of MIC WT, MIC ESR1-Y541S^homo^, and MIC ESR1-D542G^homo^ mice. (**B**–**D**) Quantification of CD3^+^ cells (total T cells), CD3^+^CD4^+^ cells (Th cells), and CD3^+^CD8^+^ cells (cytotoxic T cells), respectively. (**E**) Fluorescent IHC for F4/80, CD206, p-Stat1, PanCK, and DAPI on endpoint mammary tumors of MIC WT, MIC ESR1-Y541S^homo^, and MIC ESR1-D542G^homo^ mice. (**F**–**H**) Quantification of F4/80^+^ cells (total macrophages), F4/80^+^CD206^+^ cells (protumorigenic macrophages), and F4/80^+^p-Stat1^+^ cells (antitumorigenic macrophages), respectively. Scale bars: 100 μm. Mean ± SEM for data calculated using 1-way ANOVA with Tukey’s multiple-comparison test.

To further investigate the transcriptomic differences between MIC WT and *ESR1^mut^* tumors, bulk RNA-seq revealed numerous differentially expressed genes (DEGs) (Figure 3, A and B). Using GO term analysis, the upregulated molecular functions and cellular processes aligned with the expectations for an active ER mutation and concur with existing data, including chromosome organization, cell cycle, and metabolic regulations (Figure 3, C and E) (5, 35–38). This further validates the activity of mutant ER in our in vivo model. Interestingly, the majority of the downregulated pathways centered around immune-mediated functions such as regulation of immune system processes, adaptive immune response, and lymphocyte activation (Figure 3, D and F, and Supplemental Figure 3, A–G). These results align with the clinical observations that ER^+^ breast cancers commonly have low tumor-infiltrating immune cells, resulting in patients with ER^+^ breast cancer responding poorly in immunotherapy trials (39–41). To validate if MIC *ESR1^mut^* tumors are indeed immune-deserted, we performed fluorescent IHC for total immune cells, T cell, and macrophage populations. Surprisingly, mutant tumors were well infiltrated with CD45^+^ immune cells, as well as total CD3^+^ T cells and F4/80^+^ macrophages (Figure 4, A, B, E, and F, and Supplemental Figure 4, A, D, and E). Further analyses on subpopulations revealed that MIC *ESR1^D542G^* tumors contained more CD3^+^ total T cells compared with MIC WT and *ESR1^Y541S^* tumors, while no significant differences were observed in CD3^+^CD4^+^ or CD3^+^CD8^+^ subpopulations (Figure 4, A–D). The levels of F4/80^+^CD206^+^ protumorigenic or F4/80^+^p-Stat1^+^ antitumorigenic macrophages were comparable between genotypes (Figure 4, E–H). MIC WT and *ESR1^mut^* tumors did not differ in the number of α-SMA^+^ fibroblasts, CD31^+^ endothelial cells, Ly6G^+^ neutrophils, or NK1.1^+^ NK cells (Supplemental Figure 4, A–G).

**Figure 5:**
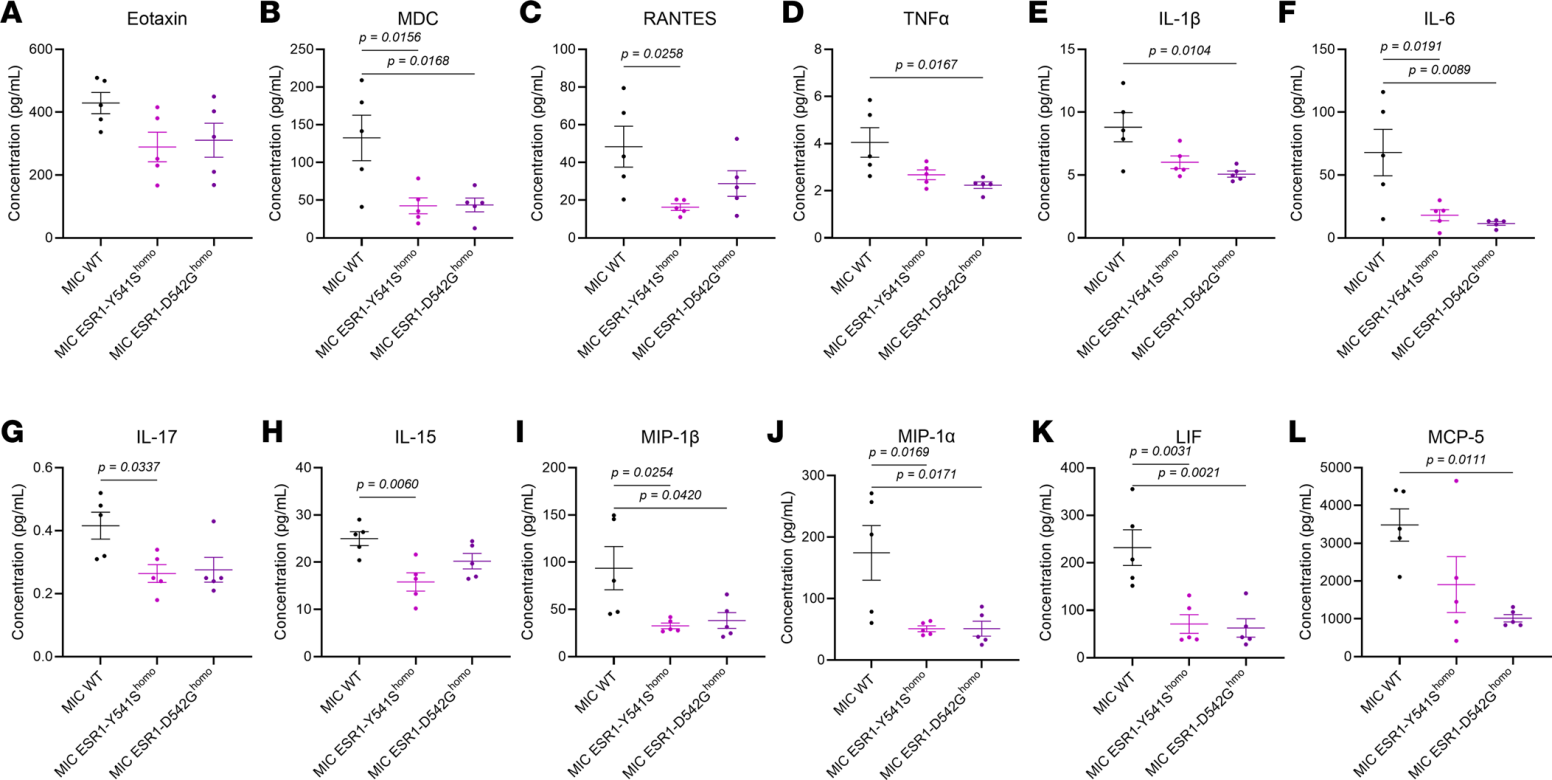
ESR1^mut^ decreases inflammatory cytokine secretion in MIC endpoint tumors. (**A**–**L**) Quantification of observed concentrations of Eotaxin, MDC, RANTES, TNF-α, IL-1β, IL-6, IL-17, IL-15, MIP-1β, MIP-1α, LIP, and MCP-5 from cytokine multiplex assay on endpoint mammary tumors of MIC WT, MIC ESR1-Y541S^homo^, and MIC ESR1-D542G^homo^ mice, respectively. Each data point represents the mean of duplicate measurements. Mean ± SEM for data calculated using 1-way ANOVA with Tukey’s multiple-comparison test.

**Figure 6:**
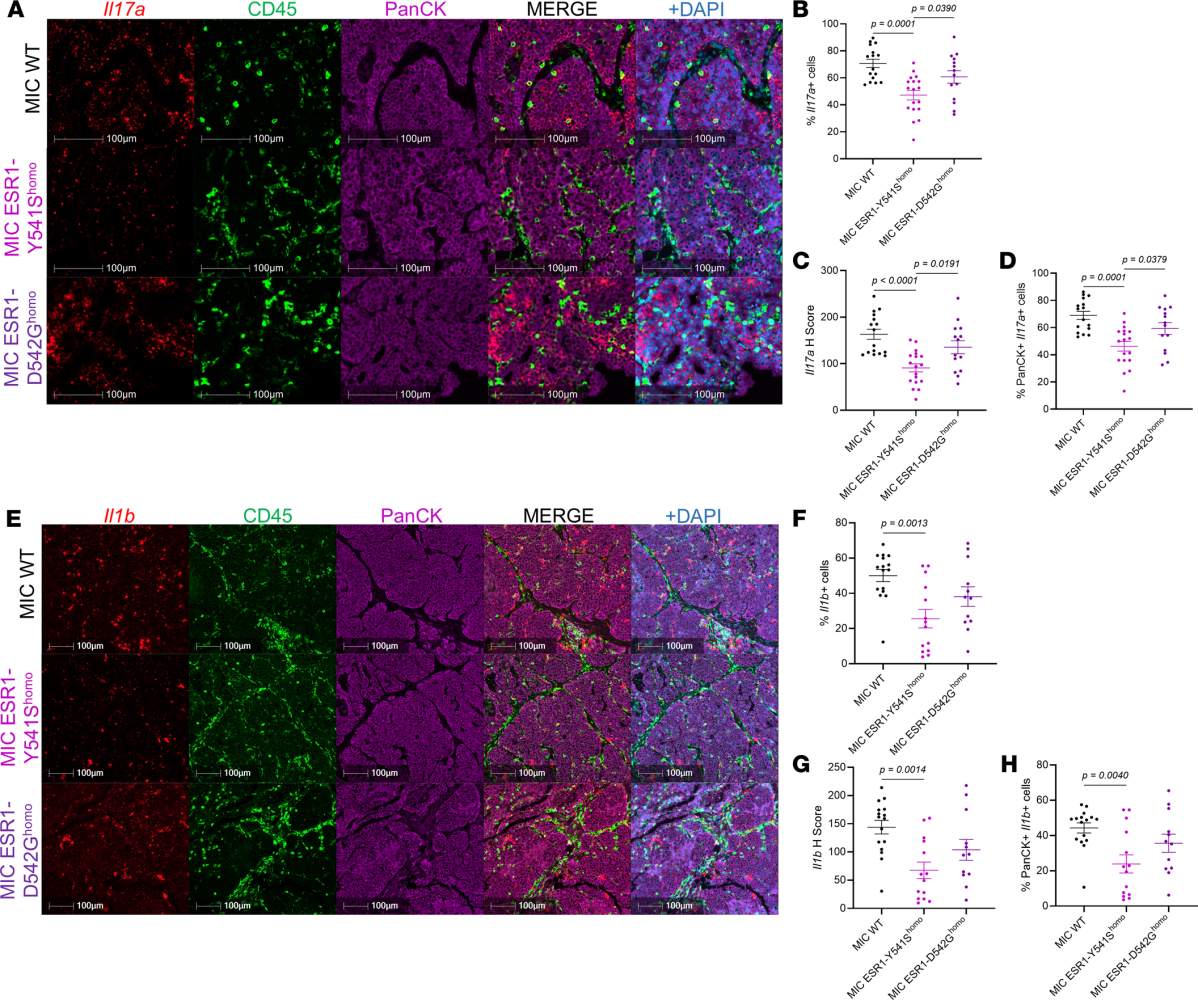
*Il17a* and *Il1b* are decreased in MIC ESR1-Y541S^homo^ endpoint mammary tumors. (**A**) RNA FISH for mouse *Il17a* and fluorescent IHC for CD45, PanCK, and DAPI on endpoint mammary tumors of MIC WT, MIC ESR1-Y541S^homo^, and MIC ESR1-D542G^homo^ mice. (**B**–**D**) Quantification of *Il17a*^+^ cells, *Il17a* H Score, and PanCK^+^ *Il17a*^+^ cells, respectively. (**E**) RNA FISH for mouse *Il1b* and fluorescent IHC for CD45, PanCK, and DAPI on endpoint mammary tumors of MIC WT, MIC ESR1-Y541S^homo^, and MIC ESR1-D542G^homo^ mice. (**F**–**H**) Quantification of *Il1b*^+^ cells, *Il1b* H Score, and PanCK^+^ *Il1b*^+^ cells, respectively. Scale bars are as indicated on each image. Mean ± SEM for data calculated using 1-way ANOVA with Tukey’s multiple-comparison test.

Although our initial histological validation results appeared to conflict with the RNA-seq data, it is well known that both the number of immune cells and their activity status are equally important indicators of their functionality and, thus, predictors of immunotherapy responsiveness (42–44). Indeed, cytokine array on tumor lysates revealed that MIC *ESR1^mut^* tumors significantly decreased their immune-modulatory cytokine levels, including TNF-α, IL-1β, IL-6, IL-17, and MIPs (Figure 5, A–L). Validation using RNA-FISH confirmed that, specifically in MIC *ESR1^Y541S^* tumors, the levels of *Il17a* and *Il1b* are significantly lower in intensity, percentage of positive cells, and colocalizing to *ESR1^Y541S^* PanCK^+^ epithelial cells (Figure 6, A–H). Moreover, although MIC *ESR1^mut^* tumors have comparable numbers of T cells and macrophages, these populations had a notable decrease in IFN-γ (*Ifng*) activity and MHC II antigen presentation, respectively (Supplemental Figure 5, A–G). Further interrogation on T cell functions in MIC *ESR1^mut^* tumors revealed that they do not alter in granzyme B, PD-1, or Tim3 levels, though MIC *ESR1^D542G^* tumors have a higher percentage of CD3^+^ CTLA4^+^ T cells (Supplemental Figure 6, A–F). Flow cytometry analyses corroborated with these results, demonstrating that MIC *ESR1^D542G^* tumors have more CD3^+^CD8^+^ cytotoxic T cells, though their levels of granzyme B and IFN-γ did not differ significantly from MIC WT tumors (Supplemental Figure 7, A, B, D, and E, and Supplemental Figure 8A). MIC *ESR1^Y541S^* tumors showed a decreasing trend in IFN-γ^+^ immune cells, with no significant differences in Lag3^+^, Tim3^+^, or TNF-α^+^CD3^+^ T cells or in TNF-α^+^CD161^+^ NK cells between the genotypes (Supplemental Figure 7, C, and F–I, and Supplemental Figure 8, A and B). Of note, while immune cells from MIC WT tumors generally showed an increase in IFN-γ positivity after PMA/ionomycin stimulation, the same trend was not observed in MIC *ESR1^mut^* tumors, indicating an impairment in activation (Supplemental Figure 7J). Additionally, MIC *ESR1^mut^* tumors did not demonstrate an increase in PD-L1 in their epithelial compartment (Supplemental Figure 7, K–M). Taken together, these results argue that, while MIC *ESR1^mut^* tumors may be adequately infiltrated with immune cells, they have dampened activity due to the decrease of *ESR1^mut^* epithelial tumor cell–derived cytokines and not because of epithelial-immune cell–mediated checkpoint inhibition by PD-L1 (45, 46).

### Mutant ER activity and upregulation of Stat5 contribute to the decreased immune cytokine secretion from ESR1^Y541S^ tumor cells.

**Figure 7:**
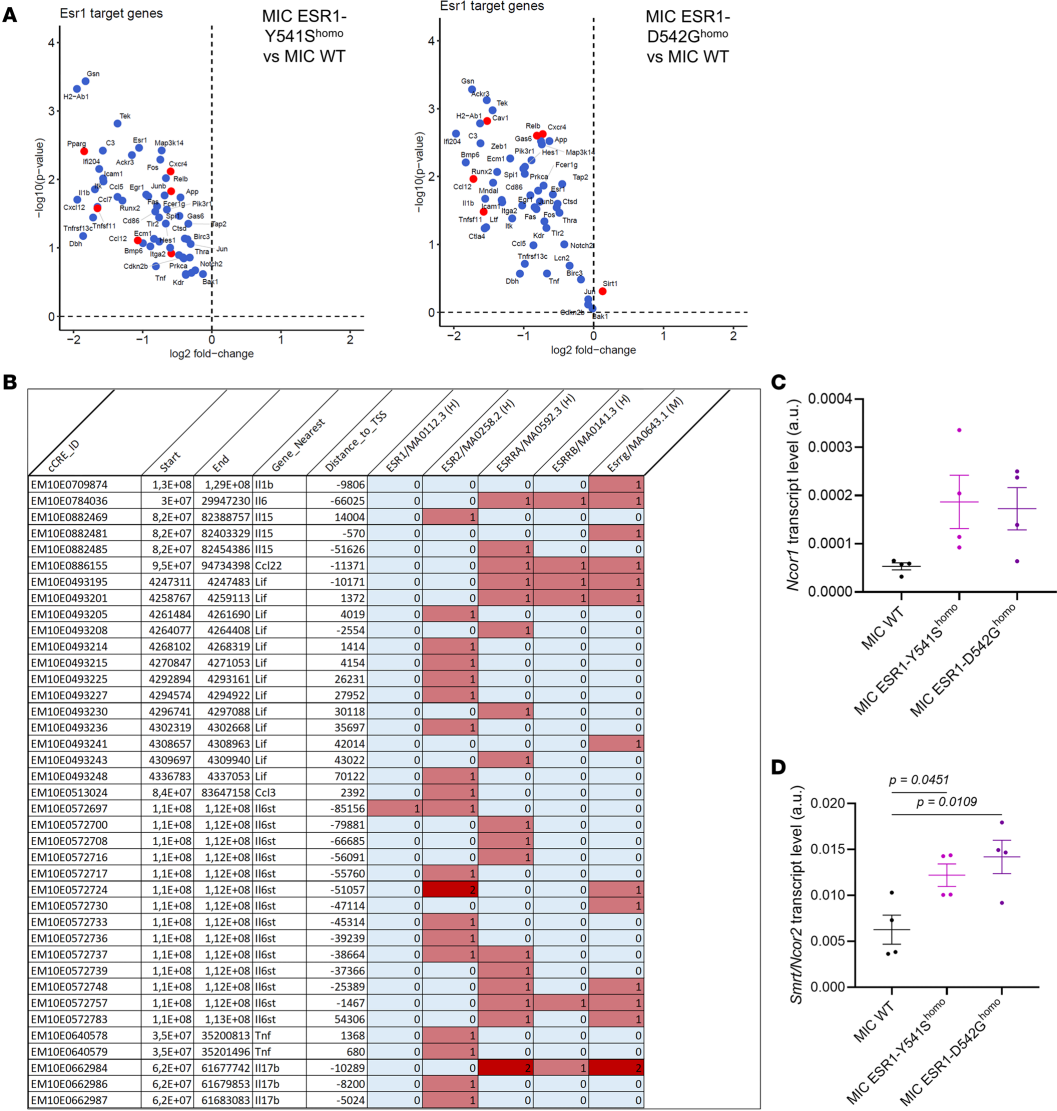
Mutant ER-target genes contribute to the decreased immune pathway activity in MIC ESR1^mut^ tumors. (**A**) Volcano plot depicting ER (*ESR1*) transcription factor-target genes contributing to the downregulated immune-related pathways in MIC ESR1-Y541S^homo^ or MIC ESR1-D542G^homo^ versus MIC WT tumors. Blue color denotes that the target genes are deactivated and concordant with the known direction of *ESR1^mut^* target interaction. Red color denotes that the target genes are deactivated but discordant to the known direction of *ESR1^mut^* target interaction. (**B**) Interrogation of estrogen response element (ERE) motifs in the promoters of downregulated immune cytokines in MIC ESR1^mut^ tumors using ENCODE as readout of their regulation by ERα. (**C** and **D**) qPCR for *Ncor1* and *Smrt*/*Ncor2* on endpoint mammary tumors of MIC WT, MIC ESR1-Y541S^homo^, and MIC ESR1-D542G^homo^ mice. Mean ± SEM for data calculated using 1-way ANOVA with Tukey’s multiple-comparison test.

Next, we sought to further elucidate the mechanism behind the phenotype where *ESR1^mut^* tumors modulate cytokine release. Previously, studies have shown that activating mutations in *ESR1* can differentially regulate ER-target gene transcription compared with WT active ER, including through the recruitment of different transcription coregulators (47–49). Indeed, analyses on the RNA-seq data reveal that *ESR1^Y541S^* and *ESR1^D542G^* target genes significantly differ from WT *ESR1*, with many downregulated genes that regulate immune-related functions such as antigen presentation (*H2-Ab1*, *Cd86*) and ILs (*Il1b*, *Tnf*, *Ccl5* [RANTES], *Ccl12* [MCP-5]) (Figure 7A). These *ESR1^mut^* downregulated genes directly contributed to the downregulated immune-modulatory pathways from GO term analysis and were seen as decreased from the immune cytokine array (Figure 3, D and F, and Figure 5). We then interrogated for estrogen response elements (EREs) in the decreased cytokines from the cytokine array and negatively regulated targets of *ESR1^mut^*, most of which were revealed to possess at least 1 ERE, indicating their regulation by an active *ESR1^mut^* (Figure 7B). Furthermore, MIC *ESR1^Y541S^* and *ESR1^D542G^* tumors showed an increase in ER corepressors NCoR1 and Smrt/NCoR2, both known to regulate ER-targeted gene transcription through protein-protein interaction with ER, supporting that *ESR1^mut^* represses immune cytokine transcription (Figure 7, C and D) (50–52).

**Figure 8:**
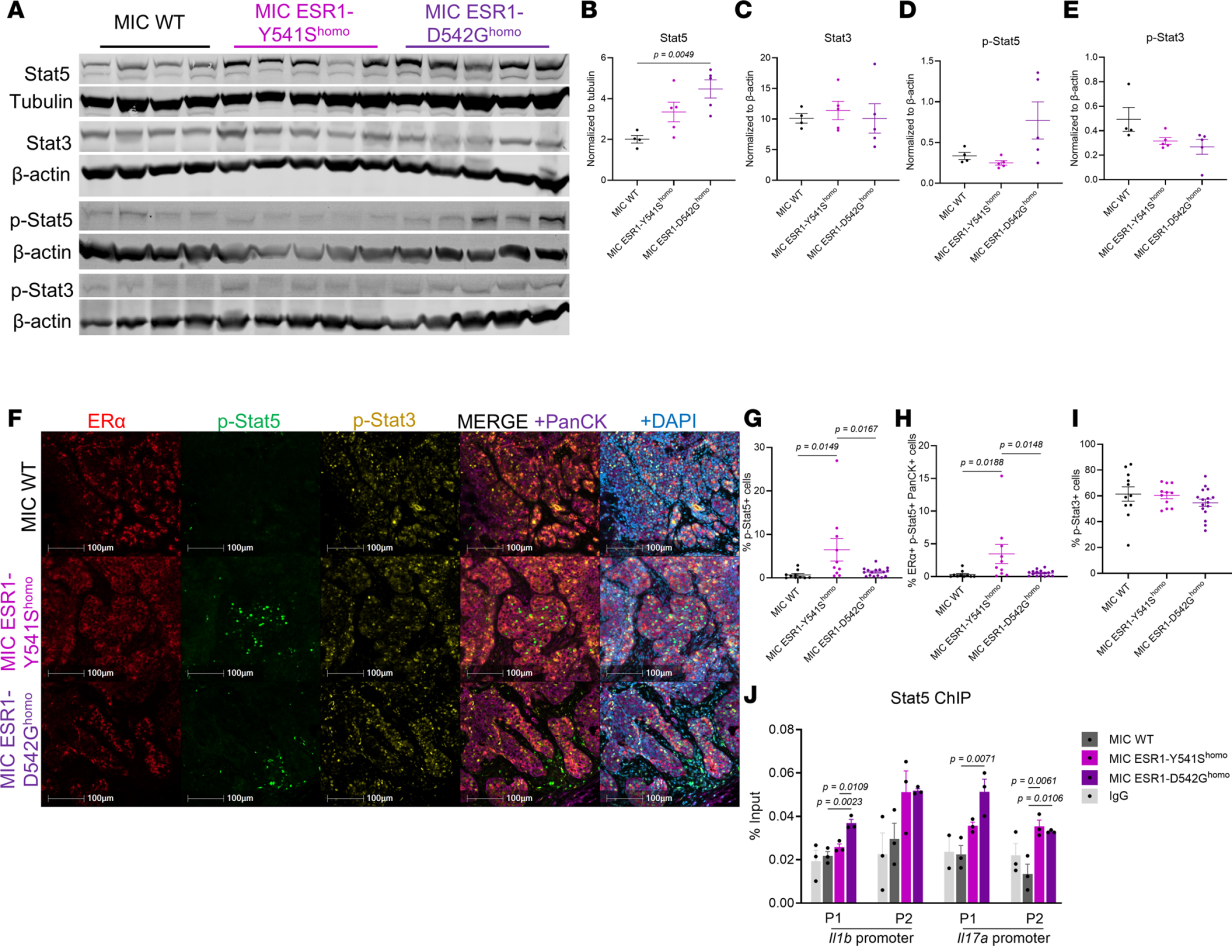
MIC ESR1-Y541S^homo^ endpoint mammary tumors have increased levels of p-Stat5, an IL-17 repressor. (**A**) Immunoblot for Stat5, Stat3, p-Stat5, and p-Stat3 with their respective loading control (Tubulin or β-actin) on endpoint mammary tumor lysates of MIC WT, MIC ESR1-Y541S^homo^, and MIC ESR1-D542G^homo^ mice. (**B**–**E**) Quantification of immunoblot for Stat5, Stat3, p-Stat5, and p-Stat3 normalized to their loading control, respectively. (**F**) Fluorescent IHC for ERα, p-Stat5, p-Stat3, PanCK, and DAPI on endpoint mammary tumors of MIC WT, MIC ESR1-Y541S^homo^, and MIC ESR1-D542G^homo^ mice. (**G**–**I**) Quantification of p-Stat5^+^ cells, ERα^+^ p-Stat5^+^ PanCK^+^ cells, and p-Stat3^+^ cells, respectively. (**J**) Stat5 enrichment on *Il1b* and *Il17a* promoter sites detected by ChIP-qPCR in endpoint mammary tumors of MIC WT, MIC ESR1-Y541S^homo^, and MIC ESR1-D542G^homo^ mice. Scale bars: 100 μm. Mean ± SEM for data calculated using 1-way ANOVA with Tukey’s multiple-comparison test.

In addition to cytokine transcription regulation directly by an active mutant ER, ER can also work in tandem with other transcription factors (53, 54). The signal transducer and activator of transcription 5 (Stat5) has been documented to interact with ER and repress Stat3-regulated immune cytokines by occupying Stat3-binding sites, thus acting as a transcriptional repressor (55, 56). Indeed, Stat5 appeared in the transcription factor network of ER (Supplemental Figure 9A). This supports that ER regulates Stat5 in addition to their direct protein-protein interaction to modulate their transcriptional regulatory functions, as demonstrated previously (54). MIC *ESR1^Y541S^* tumors showed elevated levels of total and p-Stat5, specifically in ER^+^PanCK^+^ epithelial populations that would harbor an active *ESR1^Y541S^* mutant (Figure 8, A, B, D, and F–H). The levels of total and phospho-Stat3 remained consistent between MIC WT, *ESR1^Y541S^*, and *ESR1^D542G^* tumors (Figure 8, A, C, E, F, and I). In tandem with *ESR1^mut^* downregulated genes, Stat5a- and Stat5b-downregulated target genes in MIC *ESR1^mut^* tumors also included immune-modulatory molecules and cytokines such as *Ccl12*, *Tnf*, *Fcgr1*, *Cxcl9*, *Il6*, and *Il2* (Supplemental Figure 9, B and C). ChIP for Stat5 coupled with qPCR at *Il1b* and *Il17a* promoter regions further validated that these cytokines are direct targets of Stat5 as their transcription repressor, as demonstrated in previous studies (Figure 8J and Supplemental Figure 9D) (56, 57). Higher levels of Stat5a and Stat5b in MIC *ESR1^mut^* tumors directly correlated with the downregulated immune-modulatory pathways from GO term analysis (Supplemental Figure 10A and Supplemental Figure 11A). Collectively, the data support that activating mutations in *ESR1* directly and indirectly, namely with Stat5, repress immune cytokine expression in tumor cells, thus contributing to an immunosuppressive tumor microenvironment with infiltrated but inactive T cells and macrophages.

### ESR1^Y537S^ tumors have decreased levels of immune cytokines in human metastatic breast cancer.

**Figure 9:**
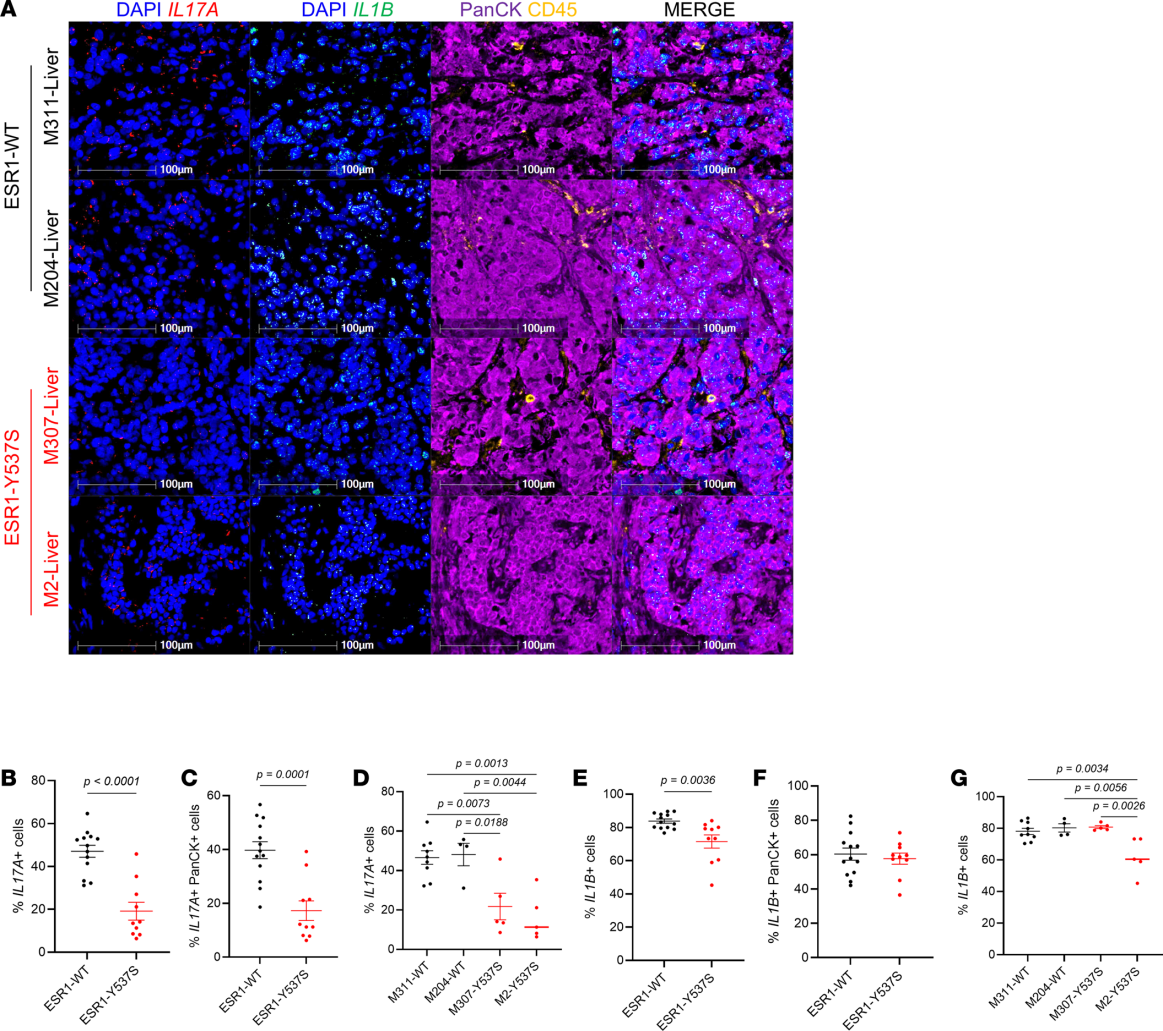
*IL17A* and *IL1B* are decreased in *ESR1^Y537S^* metastatic tumors from patients with breast cancer. (**A**) RNA FISH for human *IL17A* and *IL1B* and fluorescent IHC for CD45, PanCK, and DAPI on ESR1-WT and ESR1-Y537S patient tumors. (**B** and **C**) Quantification of *IL17A*^+^ cells and *IL17A*^+^PanCK^+^ cells by ER mutation status, respectively. (**D**) Quantification of *IL17A*^+^ cells by patient. (**E** and **F**) Quantification of *IL1B*^+^ cells and *IL1B*^+^PanCK^+^ cells by ER mutation status, respectively. (**G**) Quantification of *IL1B*^+^ cells by patient. Scale bars: 100 μm. Mean ± SEM for data calculated using 2-tailed Student’s *t* test for **B**, **C**, **E**, and **F**. Mean ± SEM for data calculated using 1-way ANOVA with Tukey’s multiple-comparison test for **D** and **G**.

To validate the clinical implications of our observations, we first interrogated an available sequencing dataset on MCF7 cell lines with or without Y537S mutation, which consistently revealed that the presence of *ESR1^Y537S^* mutation decreased several immune-related processes and pathways (Supplemental Figure 12A). Cytokine array on T47D-WT, -*ESR1^Y537S^*, and -*ESR1^D538G^* human breast cancer cell lines also showed decreases in cytokine levels, including Eotaxin, TNF-α, IL-5, and IL-1RA (Supplemental Figure 12, B–E). Of note, cytokines such as IL-17a and IL-1β could not be reliably quantified, as their levels were below the detectable range for analysis. Next, we analyzed *ESR1^WT^* or *ESR1^Y537S^* tumor samples from 4 patients with hormone receptor positive breast cancer, including lung, lymph node, uterus, and liver (Supplemental Figure 12F). All samples were pathologically evaluated to delineate malignant tumor regions (Supplemental Figure 12F). Using probes targeting human *IL17B* or *IL1B*, RNA FISH staining and analyses on signal positivity in tumor regions revealed that the levels of both cytokines were significantly lower in *ESR1^Y537S^* patient tumors, whether clustered by mutation or by patient, with most IL signals originating from the PanCK^+^ epithelial population that would contain *ESR1^Y537S^* mutation (Figure 9, A–G). Further characterization of their immune populations showed no differences in CD3^+^ T cells or CD68^+^ macrophages (Supplemental Figure 13, A–D). While the levels of p-Stat5 and p-Stat3 did not significantly differ, *ESR1^Y537S^* patient tumors had more ER positivity (Supplemental Figure 13, E–G). These data suggest that, in this particular set of patients, the driving mechanism of decreased cytokines is attributed to the presence of *ESR1^Y537S^* as the transcription repressor in tumor cells. This valuable and independent set of analyses corroborates our in vivo observations that the presence of mutant ER decreases immune cytokines secreted by tumor cells, contributing to an immunosuppressive tumor environment.

## Discussion

Patients with ER^+^ breast cancer are often given a better prognosis and 5-year survival, given that this subtype is on the less aggressive end of the malignancy spectrum and that patients have access to several endocrine therapy options targeting or modulating the ER pathway (58). However, these patients also hold the highest probability of relapse, especially latent recurrences (12, 59). Somatic activating mutation in ER/*ESR1* is a well-documented adaptation for ER^+^ tumors to overcome endocrine therapies and gain metastatic advantages (9–11). ER mutant tumors are generally resistant to traditional endocrine therapies, while the disease often progresses on chemotherapy and cyclin-dependent kinase inhibitors (8, 9, 60–62). Even with the exhaustion of the aforementioned options, these patients respond poorly to immunotherapies, as they are generally ineffective for hormone receptor–positive breast cancers, with consistently low tumor-infiltrating immune cells and limited clinical trials that include ER^+^ patients (19, 22, 41, 63). Additionally, limited in vivo studies have investigated the effect of mutant ER on the tumor immune environment (TIME) and its implications on the observed clinical manifestations.

Here, we employed the well-characterized luminal transgenic mouse model (MIC) to knock-in *ESR1^Y541S^* or *ESR1^D538G^* mutation in PyV mT–driven malignant mammary epithelial cells (23, 26). The results demonstrate that *ESR1^mut^* tumors show activation in the ER pathways without upregulating *ESR1* transcripts, and MIC *ESR1^Y541S^* mice have larger lung metastatic lesions, reflective of its clinical implications in metastatic progression. Of note, the decrease in hyperplasia in MIC *ESR1^Y541S^* mammary glands at the early time point and delayed tumor growth were unexpected. We postulate that the active mutant *ESR1^Y541S^* may be driving luminal differentiation, thus hindering malignant transformation and tumor growth—and more potently so than *ESR1^D538G^*. While we acknowledge that the MMTV promoter could be influenced by hormones, this study is controlled by MIC WT mice, which would take into account any endogenous hormone-induced variations (24, 26). Additionally, we have previously shown that *ESR1^Y541S^* alone is insufficient to drive mammary transformation, malignancy, and metastasis in vivo: the requirement for our knock-in construct in a PyV mT–driven luminal model (28).

As the field of cancer immunology continues to advance, not only is the number of tumor-infiltrating immune cells important, but their spatial distribution and activity are indicative of immunotherapy response (42, 64). While screening for traditional markers like PD-1 on T cells is an adequate indicator of treatment response, it may not suffice as a global indicator of the long-term effectiveness of immunotherapies (65). Indeed, we show that *ESR1^mut^* tumors have decreased immune-modulatory pathway activities, with a notable decrease in cytokine secretion, including IL-17a and IL-1β, IFN-γ^+^ T cells, and antigen-presenting macrophages, all while the total number of immune cells remains relatively consistent. Interestingly, MIC *ESR1^mut^* tumor–infiltrated T cells did not exhibit a significant reduction in granzyme B positivity, suggesting impaired IFN-mediated priming and overall activation, without specifically compromising granzyme-dependent cytotoxic function (66–68). Furthermore, the increase in CTLA4^+^ T cells in MIC *ESR1^D542G^* tumors may reflect selective activation and exhaustion, suggesting that a subset of patients with *ESR1^mut^* breast cancer could still benefit from specific immune checkpoint inhibitors (69). Altogether, this yields an immune-infiltrated but inactive TIME because of dampened cytokine release from *ESR1^mut^* tumor cells, resulting in a pseudo-immune hot TIME. Therefore, while assessing the tumor mutational burden during immunotherapy screens, it would be equally important to evaluate activating somatic mutations in tumor cells and their implications on the TIME, as they can significantly affect treatment outcomes.

Further investigation reveals that an active *ESR1^mut^* directly and indirectly suppresses these cytokines compared with WT *ESR1*, the latter through a Stat5-dependent mechanism. IL-17a is a potent cytokine, with Stat5 known as its repressor (70, 71). Previous studies demonstrated that Stat5 prevents Stat3 from binding to its transcription regulatory region, thus dampening Stat3 targets such as IL-17a (54, 56, 57). Consistently, we show that MIC *ESR1^Y541S^* tumors have elevated levels of p-Stat5 in epithelial cells and that Stat5 directly interacts with both *Il17a* and *Il1b* promoters. However, the relative contributions of *ESR1^Y541S^* versus Stat5 as repressors of immune modulators and their influence on the metastasis, recurrence, and therapy outcomes are beyond the scope of this study and require further investigation.

Validation on *ESR1^mut^* breast cancer cell lines demonstrates a similar decrease in cytokine profiles and characterization of *ESR1^Y537S^* patient samples confirms our in vivo observations with a significant decrease in *IL17A* and *IL1B* stemming from the epithelial compartment in malignant regions compared with WT *ESR1* patient tumors. This independent set of clinical data indicates that, while the MIC model is relatively more immunogenic than luminal patient tumors, it remains a valuable platform that adequately recapitulates human disease. A limitation in the analyses of the TIME in patients is the inability to identify which tumor cell carries the *ESR1^Y541S^* mutation, as the current detection method relies on digital droplet PCR (ddPCR). ER mutant–specific probes could provide valuable spatial information in the tumor immune landscape changes conferred by *ESR1^mut^* tumor cells. We further acknowledge the clinical heterogeneity arising from the coexistence of WT and mutant *ESR1*, as well as multiple mutants within the same tumor (72). The development, characterization, and functional dissection of additional models to better reflect these variations warrant further investigation, though they are beyond the scope of this current study.

**Figure 10:**
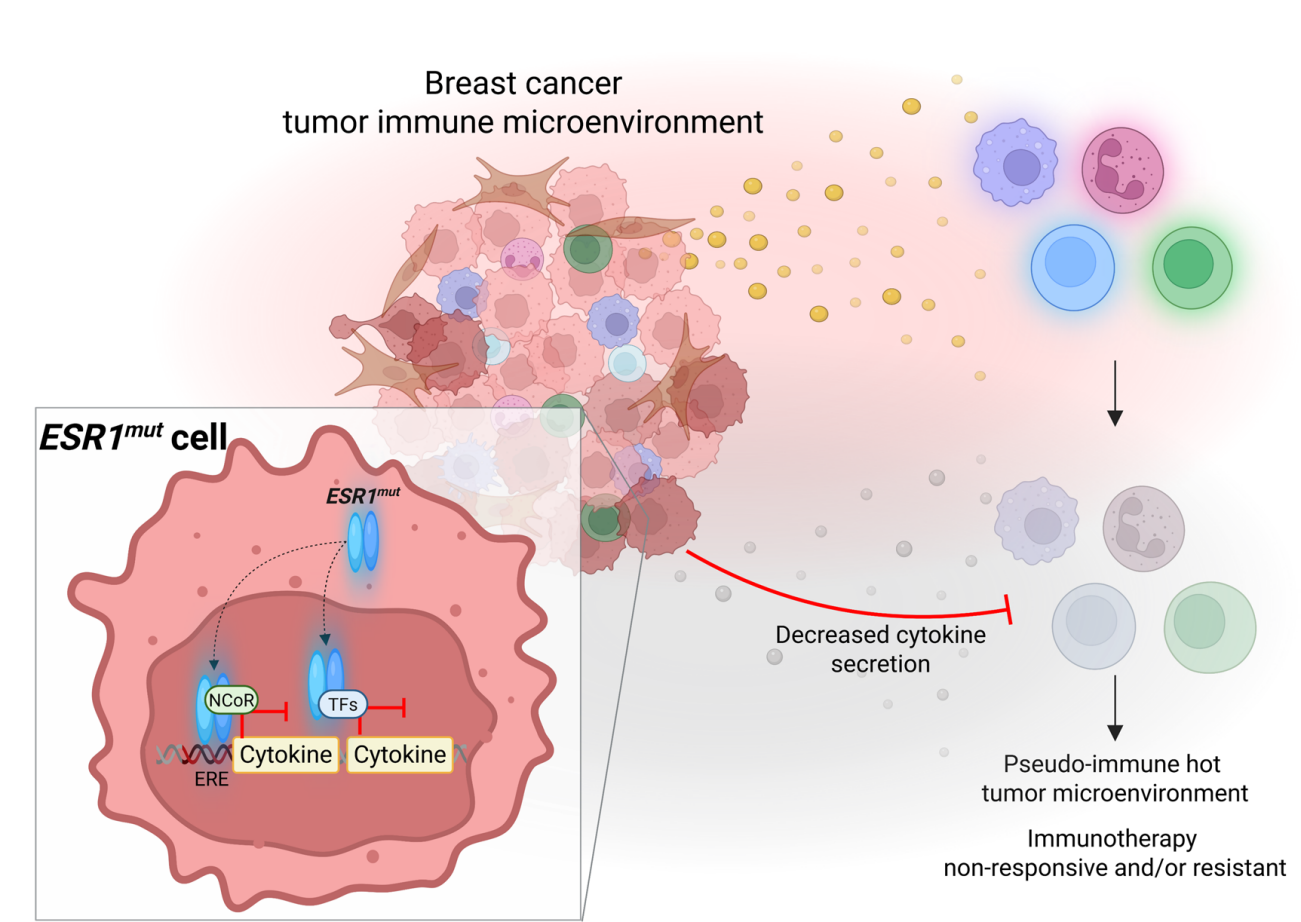
Activating mutations in *ESR1* contribute to an immunosuppressive breast tumor microenvironment by dampening cancer cell–derived cytokine secretion. (**A**) Schematic model depicting *ESR1* mutation in breast cancer cells dampens immune-modulatory cytokine secretion, resulting in an immunosuppressive breast tumor microenvironment with decreased tumor-infiltrating immune cell activity. Created in BioRender.

It is well documented that the tyrosine-to-serine mutation is more potent than the aspartic acid-to-glycine mutation, including in its activity, Ser118 phosphorylation, stability, and transcription comodulator recruitment, providing an explanation for MIC *ESR1^Y541S^* tumors to more robustly suppress the TIME (14, 73–75). We also recognize and acknowledge that numerous studies on *ESR1^mut^* have demonstrated contrasting results, where ER mutations drive a basal-like phenotype with increased immune signatures (76, 77). While many of these studies were done in vitro, by tail vein, xenografts, or orthotopic transplants, this study employs an immunocompetent in vivo system that mimics the full and natural disease progression course of breast cancer with ER mutation knocked in in an inducible and controlled manner. Furthermore, result variations would be expected between different patient sample types and locations, such as plasma versus biopsies. Therefore, due to inherent differences in models and tissue sources, variability in outcomes is anticipated. Taken together, our results argue that activating mutations in *ESR1* differentially regulate immune-modulatory gene expression to create an immunosuppressive TIME, providing insights into the clinical manifestation of their poor response to immunotherapies (Figure 10). These results deliver an additional treatment opportunity where new generations of ER mutant–sensitive endocrine therapies may carry immunotherapy sensitization potential.

## Methods

### Sex as a biological variable.

This study takes into consideration that breast cancer predominantly affects women, although we acknowledge that 1% of cases occur in men. Therefore, our study solely used female experimental mice to recapitulate breast cancer in women (biological attribute). All patient samples in this study are from women.

### Animal model.

MMTV-reverse tetracycline transactivator (rtTA) transgenic mice were generated in the laboratory of Lewis Chodosh and the generation of Tet-ON-PyV mT–IRES-Cre transgenic mouse (MIC) were previously described (23, 26). The MIC mouse was crossed to a knock-in *ESR1^Y541S^* or *ESR1^D542G^*-mutant exon 9. All mice were maintained on the pure FVB background. Genomic DNA was extracted from tails of all mice using crude salt extraction and subsequently used for genotype confirmation using PCR described previously for MMTV-rtTA and for MIC (78). Genotype confirmation for floxed *ESR1^mut^* was done using the following primers (5′–3′): *ESR1^Y541S^* forward: CGCCCCATATTTTGAACACAG; *ESR1^Y541S^* reverse: ACGAGTATGGAGAGTGTCAGG; *ESR1^D542G^* forward: GACTGTGCCTTCTAGTTGCC; *ESR1^D542G^* reverse: CATCTCCAGGAGCAGGCCAT. Experimental and control animals were given drinking water with doxycycline (2 mg/mL) at 8–12 weeks of age (“induction”) and monitored weekly by physical palpation for tumor formation.

### Mouse tissue collection.

Mammary gland, mammary tumor, lung, and liver were collected at various time points throughout this study. All tissues collected were analyzed unless tissue quality was deemed insufficient. Mice were euthanized at endpoint, when an individual tumor or the total tumor mass reached the endpoint burden defined by McGill Animal Ethics Guidelines, or at various experimental or treatment endpoints. All solid organ tissues were fixed for 36 hours in 10% (vol/vol) formalin (Leica), embedded in paraffin, and sectioned at 4 μm for histological staining or were flash frozen in liquid nitrogen and kept at –80°C for immunoblot, RNA extraction, or cytokine array sample preparation. Mammary gland for whole mount analyses were incubated in acetone for 24 hours, stained in hematoxylin for 24 hours, destained in 70% ethanol with 1% HCl, dehydrated in 70% and 100% ethanol, and in xylenes until mounting. Slides were scanned using the ZEISS Axio Zoom.V16 microscope. H&E staining was performed by the Histology Innovation Platform at the Goodman Cancer Institute. H&E slides were scanned using the Nanozoomer S210 at 20X and analyzed using HALO 2.0 software (Indica Lab).

### Human cell lines.

T47D cell lines, WT and *ESR1^mut^*, were obtained from the Lee/Oesterreich laboratories, from the Department of Pharmacology and Chemical Biology, University of Pittsburgh, and the Women’s Cancer Research Center, Magee-Womens Research Institute, UPMC Hillman Cancer Center. Individual clones were used for cytokine array, as described previously (76). Cells were maintained in RPMI with 10% FBS, 0.2% human insulin, 100 μg/mL penicillin, and 100 mg/mL streptomycin at 37°C with 5% CO_2_.

### Fluorescent IHC, imaging, and quantitative analysis.

Sample collection and preparation for paraffin-embedded mouse tissues were described above. Fluorescent IHC was performed as previously described (79). Stained slides were scanned using the Axio Scan Z1 digital slide scanner (Carl Zeiss) and analyzed using HALO 4.1 software (Indica Lab).

The following antibodies were used for fluorescent IHC on mouse tissue: ERα (Abclonal, A12976, 1:500), PyV mT (Santa Cruz, 53481, 1:200), PanCK (Ventana, 760-2135, 1:10), α-SMA (DAKO, M0851, 1:400), CD31 (CST, 77699, 1:100), CD45 (CST, 70257, 1:200), Ly6G (CST, 87048, 1:100), NK1.1 (CST, 39197, 1:200), CD3 (Abcam, ab16669, 1:200), CD4 (CST 25229, 1:50), CD8 (CST, 98941, 1:200), F4/80 (CST, 70076, 1:200), CD206 (CST, 24595, 1:400), p-Stat1 (CST, 9167, 1:200), MHC II (BD, 107601, 1:200), Granzyme B (CST, 44153, 1:200), PD-1 (CST, 84651, 1:200), PD-L1 (CST, 64988, 1:100), Tim3 (CST, 83882, 1:200), CTLA4 (CST, 53560, 1:100), p-Stat5 (CST, 9314, 1:100), and p-Stat3 (CST, 9145, 1:100).

The following antibodies were used for fluorescent IHC on human tissue: CD45 (Ventana, 760-4279, 1:10), PanCK (Ventana, 760-2135, 1:10), CD3 (Abcam, Ab16669, 1:200), CD68 (Ventana, 790-2931, 1:10), ERα (Ventana, 790-4324, 1:10), p-Stat5 (Abcam, ab32364, 1:50), and p-Stat3 (CST, 9145, 1:50).

### RNA extraction.

Flash-frozen pieces of tumors were crushed in liquid nitrogen. Total RNA was isolated using FavorPrep Tissue Total RNA Mini Kit (Cat Number FATRK 001) according to manufacturer’s protocol. RNA quantity was determined using NanoDrop Spectrophotometer ND-1000 (NanoDrop Technologies Inc.). cDNA was synthesized by reverse transcription using the TranScript all-in-one first strand cDNA synthesis kit (TransGen Biotech) for qPCR.

### qPCR analysis.

qPCR was performed using LightCycler 480 SYBR Green I Master Reagents (Roche). Data were normalized to *Gapdh* to generate the relative transcript levels using the expression 2^(crossing^ ^point^ ^value^ ^of^ *^Gapdh^* ^−^^crossing^ ^point^ ^value^ ^of^ ^gene^ ^of^ ^interest)^. Each reaction was run in triplicate. The following primers were used for qPCR analysis (5′–3′): *Ncor1*, left primer: CTGGTCTTTCAGCCACCATT, right primer: CCTTCATTGGATCCTCCATC; *Smrt/Ncor2*, left primer: ATGGCTTGTCTGAGCAGGAG, right primer: GGGTCATCCATGAGTCCATT; *Gapdh*, left primer: CTGCACCACCAACTGCTTAG, right primer: GTCTTCTGGGTGGCAGTGAT.

### RNA-seq and analysis.

Library construction, quality assessment and sequencing (Illumina NovaSeq Paired-End 100 base pair – 25 million reads) were performed by Génome Québec on endpoint tumor total RNA (MIC *n* = 3, MIC *ESR1^Y541S^* *n* = 3, MIC *ESR1^D542G^* *n* = 3). Data analyses, including DEGs and GO enrichment, were performed by Alain Pacis from the Canadian Centre for Computational Genomics. For heatmap and hierarchical clustering, DEGs with *P* values adjusted for multiple testing (FDR) < 0.05 were clustered for similar expression using standardized FPKM values (*z* scores).

### RNA FISH.

RNA FISH was performed on paraffin-embedded tissue sections using RNAscope 2.5 HD Detection Reagents-RED (ACD, #322360) for mouse tissue and RNAscope Multiplex Fluorescent Detection Kit v2 (ACD, #323110) for human tissue, respectively, according to the manufacturer’s protocol. Stained slides were scanned using the Axio Scan Z1 digital slide scanner (Carl Zeiss) and analyzed using HALO 2.0 software (Indica Lab).

The following probes were used on mouse tissue: Mm-Esr1 (REF 478201), Mm-Il17a (REF 319571), and Mm-Il1b (REF 316891). The following probes were used on human tissue: Hs-IL17A (REF 310931-C3) and Hs-IL1B (REF 310361-C4). Subsequently, fluorescent IHC was performed according to the protocol above.

### Immunoblot sample preparation and analysis.

Flash-frozen mammary gland or tumor pieces were crushed in liquid nitrogen, and cells were collected into Eppendorf tubes by scraping and rinsed once in 1X PBS. From here, both sample types are treated equally as previously described (79). Briefly, samples were incubated in lysis buffer (10 mM Tris-HCl [pH 8.0], 1 mM EDTA, 0.5 mM EGTA, 1% Triton X-100, 0.1% sodium deoxycholate, 0.1% sodium dodecyl sulfate, 140 mM sodium chloride, 2 mM sodium pyrophosphate, 5 mM sodium fluoride, 10 mM β-glycerophosphate) with protease inhibitors (AEBSF 50 μg/mL, aprotinin 10 μg/mL, Leupeptin 10 μg/mL, Na_3_VO_4_ 100 μg/mL) for 1 hour rotating at 4°C, centrifuged at maximum speed (16,000*g*) for 15 minutes, and supernatant was collected. The protein concentration in supernatant was determined from OD reading and calculated in reference to a BSA standard curve. Equal quantity of protein per sample was loaded on acrylamide gel for running then transferred onto Immobilon-FL PVDF transfer membrane. Membranes were blocked using Li-Cor Odyssey Blocking Buffer (TBS), incubated in primary antibodies overnight at 4°C, washed in TBS with 1% Triton X-100, incubated in secondary antibodies (1:10,000) for 1 hour at room temperature, washed, and imaged using Sapphire FL Biomolecular Imager. Band intensity quantification was done using Image Studio Lite software (Li-Cor).

The following antibodies were used for immunoblots: β-actin (Sigma, A5441, 1:2000), tubulin (Cell Signaling Technology (CST), 2148, 1:1000), Cyclin D1 (CST, 2922, 1:500), FOXA1 (Abcam, ab23738, 1:1000), Stat3 (CST, 9139, 1:1000), Stat5 (CST, 94205, 1:1000), p-Stat3 (CST, 9145, 1:1000), and p-Stat5 (CST, 4322, 1:500).

### Cytokine array.

Flash-frozen tumor pieces were treated as an immunoblot sample preparation for tumor lysate. For adherent cell lines, cells were washed in ice-cold 1X PBS, scraped into Eppendorf tubes, and pelleted. The pellets were resuspended in lysis buffer as described above and treated as an immunoblot sample preparation. Tumor lysates and cell lysates were prepared according to the manufacturer’s protocol and shipped to Eve Technologies for analysis. At least duplicates were run for each sample.

### ChIP-qPCR.

Around 40 mg of flash frozen tissues were minced in liquid nitrogen and resuspended in ice-cold 1X PBS with protease inhibitors (AEBSF 50 μg/mL, aprotinin 10 μg/mL, Leupeptin 10 μg/mL, Na_3_VO_4_ 100 μg/mL), manually homogenized using a Dounce Homogenizer, and strained through a 70 mm strainer. The samples were crosslinked for 10 minutes at room temperature with 1% formaldehyde and quenched with 0.125M glycine for 8 minutes. The pellets were washed twice with 1X PBS and incubated in lysis buffer (10 mM Tris-HCl pH 8.0, 10 mM EDTA, 1 mM EGTA, 0.25% Triton X-100, with protease inhibitors) for 15 minutes at room temperature. The pellets were washed in lysis wash buffer (10 mM Tris-HCl pH 8.0, 200 mM NaCl, 10 mM EDTA, 0.5 mM EGTA, with protease inhibitor) for 10 minutes at 4**°**C and resuspended in 600 μL of sonication buffer (10 mM Tris-HCl pH 8.0, 10mM EDTA, 0.5 mM EGTA, 0.1% SDS, with protease inhibitor). After optimized sonication and centrifugation at 14,000*g*, the supernatant was precleared with 25 μL Dynabeads Protein A (Invitrogen, 10001D). Immunoprecipitation was performed with 500 μL precleared supernatant using Stat5 antibody (CST, 94205, 0.157 mg/mL) or IgG control (CST, 2729, 1 mg/mL) and 500 μL dilution buffer (16.7 mM Tris-HCl pH 8.0, 150 mM NaCl, 1.2 mM EDTA, 1.1% Triton X-100, 0.01% SDS) overnight at 4**°**C, 40 μL supernatant as input. In total, 50 μL of beads were added and incubated at 4**°**C for 2 hours. Beads were washed 3 times with wash buffer I (50 mM HEPES pH 7.9, 150 mM NaCl, 1 mM EDTA, 1% Triton X-100, 0.1% SDS, 0.1% deoxycholate), twice with wash buffer II (50 mM HEPES pH 7.9, 500 mM NaCl, 1 mM EDTA, 1% Triton X-100, 0.1% SDS, 0.1% deoxycholate), and twice with TE buffer (10 mM Tris-HCl pH 8.0, 1 mM EDTA). The beads were eluted twice with 100 μL elution buffer (1% SDS, 50 mM Tris-HCl pH 8.0, 1 mM EDTA) at 65**°**C. Protease K was added to the elution and input, incubated at 65°C overnight, and purified with MinElute PCR Purification Kit for PCR Cleanup according to manufacturer’s protocol (Qiagen, 28004). Purified DNA was used for qPCR analysis as described above. The following primers were used (5′–3′): *Il1b* promoter-1 left primer: CTCAGGTGCCATGTGTCCAT, right primer: AGAAATAGAGGTGGGAGGACA; *Il1b* promoter-2 left primer: TCTCAGACTTCTTTGTTTCGCT, right primer: GGAACGAGACTCTGACCTAAAA; *Il17a* promoter-1 left primer: AGCTCCCAAGAAGTCATGCT, right primer: ATGAGGTCAGCACAGAACCA; *Il17a* promoter-2 left primer: GTGAAAGTCAGAGTTACCAGCC, right primer: CCGTGTTCTCAGAAGTTGCA.

### Flow cytometry on mouse mammary tumors.

Mammary tumors were dissociated into a single-cell suspension. Briefly, each tumor sample was chopped using the McIlwain Tissue Chopper and incubated in 10 mL of dissociation media (collagenase IV 3.125 mg/mL, collagenase I 0.8 mg/mL, DNase I 0.1 mg/mL) for 30 minutes, rotating at 37°C, and centrifuged for 10 minutes at 600*g*. Red blood cells were lysed using ACK lysis buffer (150 mM NH_4_Cl, 10 mM KHCO_3_, 0.1 mM Na_2_EDTA, pH 7.5) and neutralized with FACS buffer (1X PBS, 2% FBS, 2 mM EDTA). The suspension was strained, centrifuged at 600*g*, and resuspended in FACS buffer. All samples were treated with BD GolgiPlug Protein Transport Inhibitor (Containing Brefeldin A) (BD, 51-2301KZ), with or without eBioscience Cell Stimulation Cocktail (plus protein transport inhibitors) PMA/ionomycin stimulation (ThermoFisher, 00-4975-93) according to manufacturers’ protocols for 5 hours at 37°C. Cell suspensions were washed in FACS buffer, blocked with Fc block TruStain FcX PLUS (anti-mouse CD16/32) (BioLegend, 156604, 1:300), stained for extracellular markers, fixed using BD Cytofix/Cytoperm Plus (BD Bioscience, #555028) according to the manufacturer’s protocol, and stained for intracellular markers.

The following antibodies were used for flow cytometry: eBioscience Fixable Viability Dye eFluor 506 (ThermoFisher, 65-0866-14, 1:1000), CD45 (BD, 564225, 1:500), CD3 (BioLegend, 100204, 1:200), CD4 (BioLegend, 100422, 1:300), CD8 (BioLegend, 100748, 1:300), IFN-γ (BioLegend, 505808, 1:400), granzyme B (BioLegend, 515408, 1:1 or 5 μL), CD223/Lag3 (BioLegend, 125224, 1:400), CD366/Tim3 (BioLegend, 119721, 1:400), TNF-α (BioLegend, 506306, 1:800), and CD161 (BD, 750399, 1:300).

### Patient sample collection and pathology evaluation.

Patient samples were obtained from the University of Pittsburgh Hope for OTHERS (HfO) program under IRB STUDY19060376 approval and with written patient consent at the Magee Womens Cancer Research Center at UPMC Hillman Cancer Center, Magee Womens Research Institute (80). Samples are formalin-fixed paraffin-embedded tissues of ER WT or ER Y537S mutant metastatic tissue samples. Y537S mutational status of the patients was confirmed with ddPCR using probe sequence (/5HEX/TC TCT GAC C/ZEN/T GCT GCT GGA GAT GCT/3IABkFQ/) from Integrated DNA Technologies, Inc, and WT confirmation using (/56-FAM/TC TAT GAC C/ZEN/T GCT GCT GGA GAT GCT/3IABkFQ/) from Integrated DNA Technologies Inc. All samples were pathologically evaluated to delineate tumor regions by Marie-Christine Guiot at the Montreal Neurological Institute-Hospital, McGill University.

### Statistics.

Sample sizes are indicated in each figure and figure legend. Sample sizes were determined based on our previous experience. No in vivo therapeutic experiments were done in this study that required randomization or blinding. Data from all experiments were analyzed, and *P* values from statistical tests were used to assess statistical significance and appropriateness of sample sizes. All statistical analyses were done using GraphPad Prism 5 software. Significance between 2 sets of data was assessed using 2-tailed Student’s *t* test. Significance between more than 2 sets of data was assessed using 1-way ANOVA with Tukey’s multiple-comparison test unless otherwise indicated. Data represent mean ± SEM for biological replication or ± SD for technical replication. For Kaplan-Meier survival analysis, statistical significance was calculated by log-rank (Mantel-Cox) test. For mammary tumor growth curves, compare groups of growth curves (CGGC) permutation test was used (81, 82). For GO enrichment analyses, the *P* value is the probability of seeing at least x number of genes out of the total *n* genes in the list annotated to a particular GO term, given the proportion of genes in the whole genome that are annotated to that GO term (83, 84). *P* < 0.05 was considered statistically significant.

### Study approval.

All mice from animal experiments in this study were housed and handled at the Comparative Medicine and Animal Resource Centre at McGill University, approved by and in compliance with the Animal Ethics Committee, Facility Animal Care Committee, and Canadian Council on Animal Care (protocol no. MCGL5518).

### Data availability.

The values for all data points in the figure panels are reported in the Supporting Data Values file. Raw and processed RNA-seq data are available on GEO repository under the accession number GSE299798.

## Author contributions

Conception and design were contributed by YG and WJM. Development of methodology was contributed by YG, DZ, QXH, VSG, AP, MCG, ACCC, TT, CL, AVL, and SO. Acquisition of data was contributed by YG, DZ, QXH, VSG, AP, MCG, ACCC, TT, CL, AVL, and SO. Analysis and interpretation of data were contributed by YG, QXH, VSG, AP, and MCG. Writing, review, and revision of the manuscript were contributed by YG and WJM. Funding acquisition was contributed by YG, SO, and WJM. Study supervision was contributed by WJM.

## Funding support

This work is the result of NIH funding, in whole or in part, and is subject to the NIH Public Access Policy. Through acceptance of this federal funding, the NIH has been given a right to make the work publicly available in PubMed Central.

- NIH (grant no. NIH RO1 CA221303-01A; SO and WJM)
- Fonds de Recherche Québec Santé and the Canadian Institutes of Health Research (no. 187660; YG)
- China Scholarship Council (no. 202406270221; QH)
- Canada Research Chair in Molecular Oncology (WJM)
- CIHR Foundation (grant no. FDN-148373; WJM)
- CCSRI innovation programs (grant no. 706679; WJM)

## Supplementary Material

Supplemental data

Unedited blot and gel images

Supporting data values

## Version 1 03/09/2026

Electronic publication
