# Supplementary material for: Activating mutations in ESR1 contribute to an immunosuppressive breast tumor microenvironment by dampening cytokine secretion
Source: JCI Insight. 2026 Mar 9;11(5):e199927. doi: 10.1172/jci.insight.199927 (PMC13041671; doi:10.1172/jci.insight.199927)
Supplement: Supplemental data [file jciinsight-11-199927-s080.pdf]

Title:

Activating mutations in *ESR1* contribute to an immunosuppressive breast tumor microenvironment by dampening cytokine secretion

Authors:

Yu Gu [1, 2], Dongmei Zuo [1], Qixin Hu [1, 3], Virginie Sanguin-Gendreau [1], Alain Pacis [4], Marie-Christine Guiot [5], Alexander Chih-Chieh Chang [6, 7], Tarek Taifour [1, 8], Chen Ling [1], Adrian V Lee [6, 7], Steffi Oesterreich [6, 7], and William J. Muller [1, 2, 9]

Supplemental Material

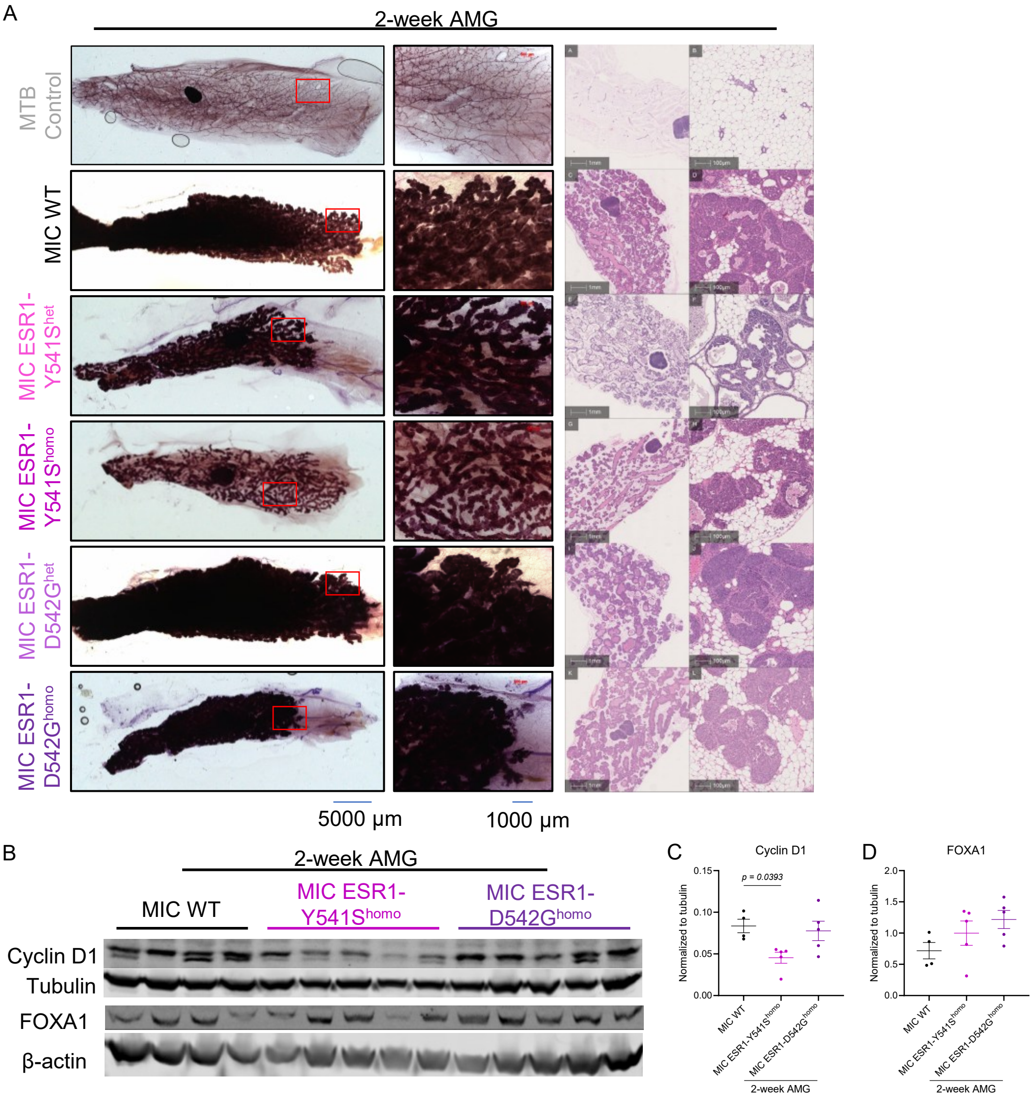

Supplemental Figure 1. Characterization of mammary glands 2 weeks post-DOX induction in MIC WT and ESR1^{mut} mice. A. Representative images of whole mount analysis and H&E images of mammary gland (AMG) epithelial transformation (tumour initiation) after 2 weeks post-DOX induction in MTB control (n = 4), MIC WT (n = 5), MIC ESR1-Y541S^{het} (n = 6), MIC ESR1-Y541S^{homo} (n = 7), MIC ESR1-D542G^{het} (n = 7), and MIC ESR1-D542G^{homo} (n = 6) mice. B. Immunoblot for cyclin D1 and FOXA1 with their respective loading control (Tubulin and β-actin) on 2 weeks post-DOX induction AMG lysates of MIC WT, MIC ESR1-Y541S^{homo}, and MIC ESR1-D542G^{homo} mice. C-D. Quantification of immunoblot for cyclin D1 and FOXA1 normalized to their respective loading control, respectively. Scale bars are as indicated on each image. Mean ± SEM for data calculated using one-way ANOVA with Tukey's multiple comparisons test.

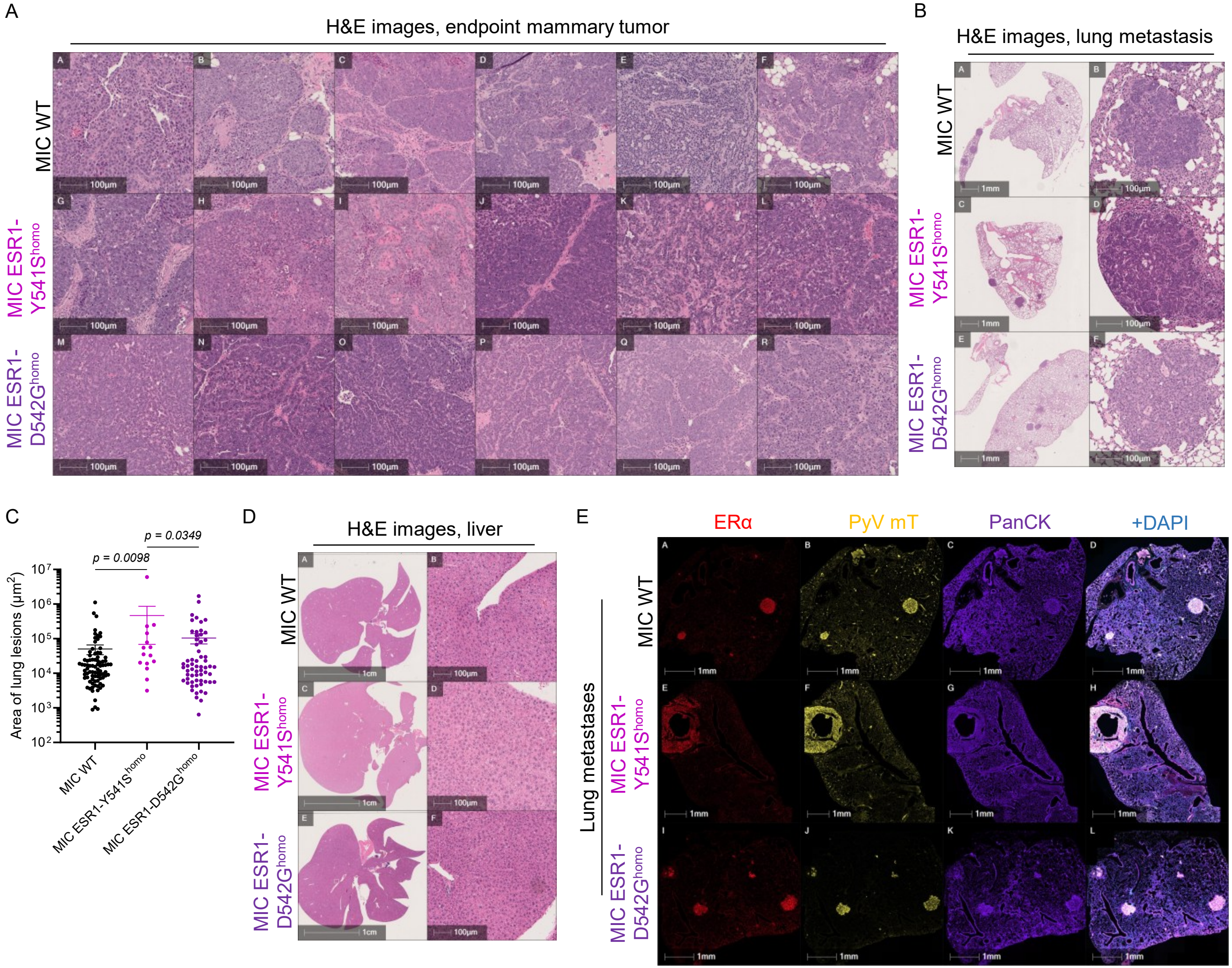

Supplemental Figure 2. Representative H&E images of endpoint mammary tumors, lungs, and liver in MIC WT and ESR1^{mut} mice. A. Representative H&E images of endpoint mammary tumors of MIC WT, MIC ESR1-Y541S^{homo}, and MIC ESR1-D542G^{homo} mice. B. Representative H&E images of lungs collected at endpoint of mammary tumor mass of MIC WT (n = 10), MIC ESR1-Y541S^{homo} (n = 6), and MIC ESR1-D542G^{homo} (n = 10) mice. C. Quantification of area of lung metastatic lesions. D. Representative H&E images of livers collected at endpoint of mammary tumor of MIC WT (n = 10), MIC ESR1-Y541S^{homo} (n = 6), and MIC ESR1-D542G^{homo} (n = 10) mice. E. Fluorescent IHC for ERα, PyV mT, PanCK, and DAPI on lung metastases of MIC WT, MIC ESR1-Y541S^{homo}, and MIC ESR1-D542G^{homo} mice. Scale bars are as indicated on each image. Mean ± SEM for data calculated using one-way ANOVA with Tukey's multiple comparisons test.

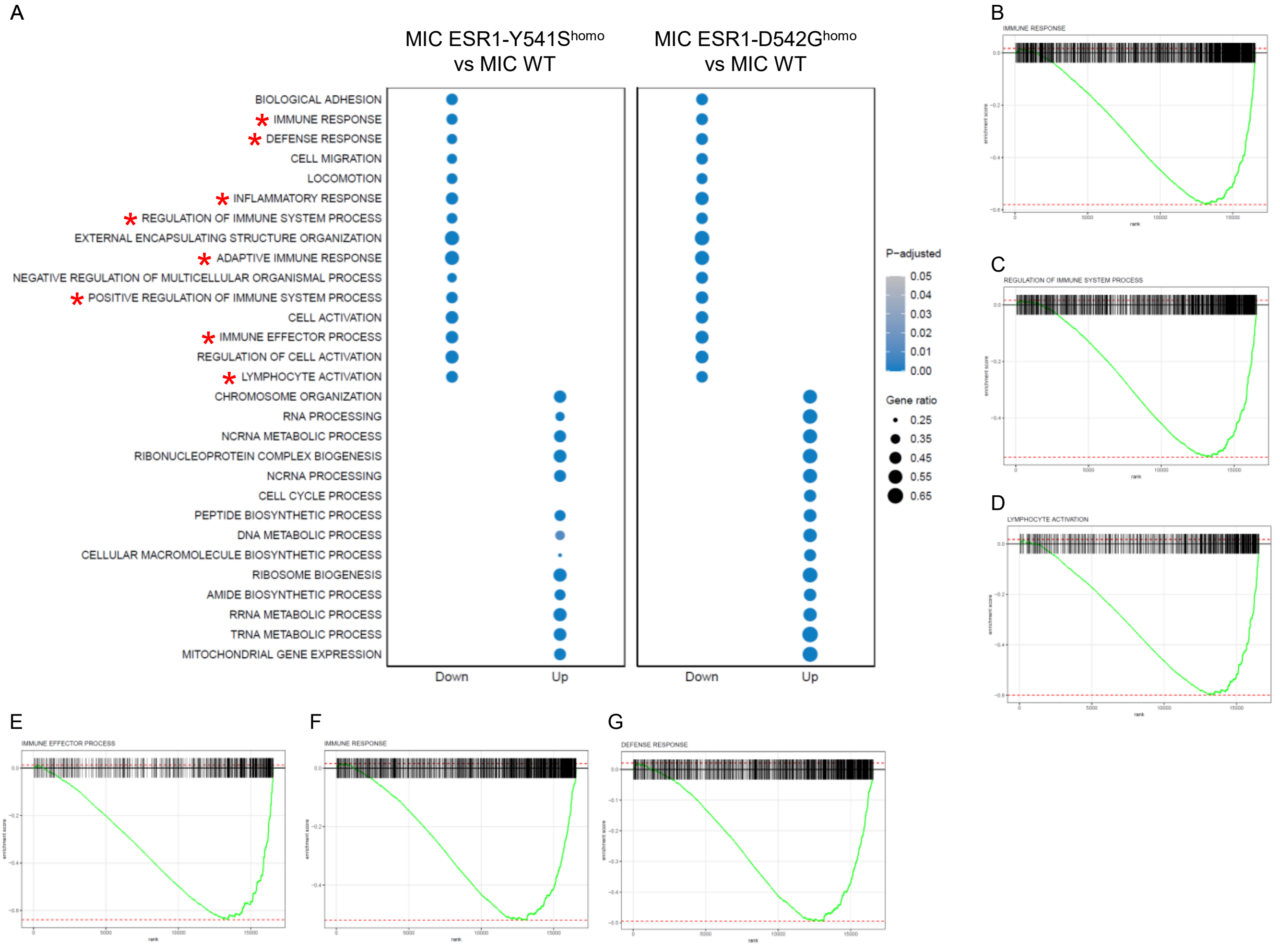

Supplemental Figure 3. GSEA analysis on MIC WT and MIC ESR1-Y541S^{homo} or MIC ESR1-D542G^{homo} endpoint mammary tumor RNA sequencing. A. Dot plots representing the changes in GSEA hallmark pathways between MIC ESR1-Y541S^{homo} tumors versus MIC WT tumors and MIC ESR1-D542G^{homo} tumors versus MIC WT tumors with stars delineating immune-related pathways. B-E. Immune response, regulation of immune system process, lymphocyte activation, and immune effector process are negatively regulated in MIC ESR1-Y541S^{homo} tumors versus MIC WT tumors, respectively. F-G. Immune response and defense response are negatively regulated in MIC ESR1-D542G^{homo} tumors versus MIC WT tumors, respectively.

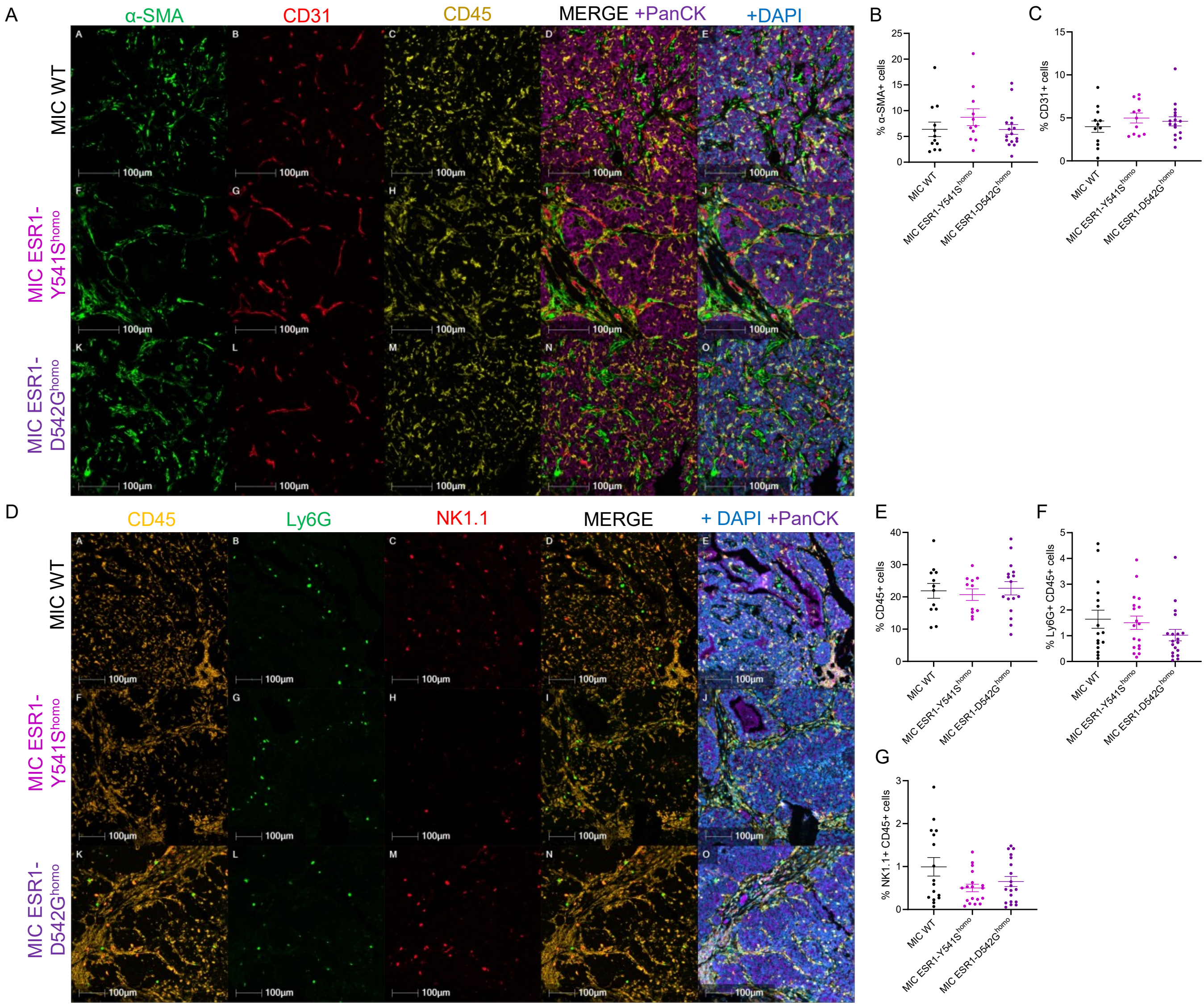

Supplemental Figure 4. Characterization of total immune cells, endothelial cells, neutrophils, and NK cells in endpoint mammary tumors. A. Fluorescent IHC for α-SMA, CD31, CD45, PanCK, and DAPI on endpoint mammary tumors of MIC WT, MIC ESR1-Y541S^{homo}, and MIC ESR1-D542G^{homo} mice. B-C. Quantification of α-SMA+ cells and CD31+ cells, respectively. D. Fluorescent IHC for CD45, Ly6G, NK1.1, PanCK, and DAPI on endpoint mammary tumors of MIC WT, MIC ESR1-Y541S^{homo}, and MIC ESR1-D542G^{homo} mice. E-G. Quantification of CD45+ cells, Ly6G+ CD45+ cells, and NK1.1+ CD45+ cells, respectively. Scale bars are as indicated on each image. Mean ± SEM for data calculated using one-way ANOVA with Tukey's multiple comparisons test.

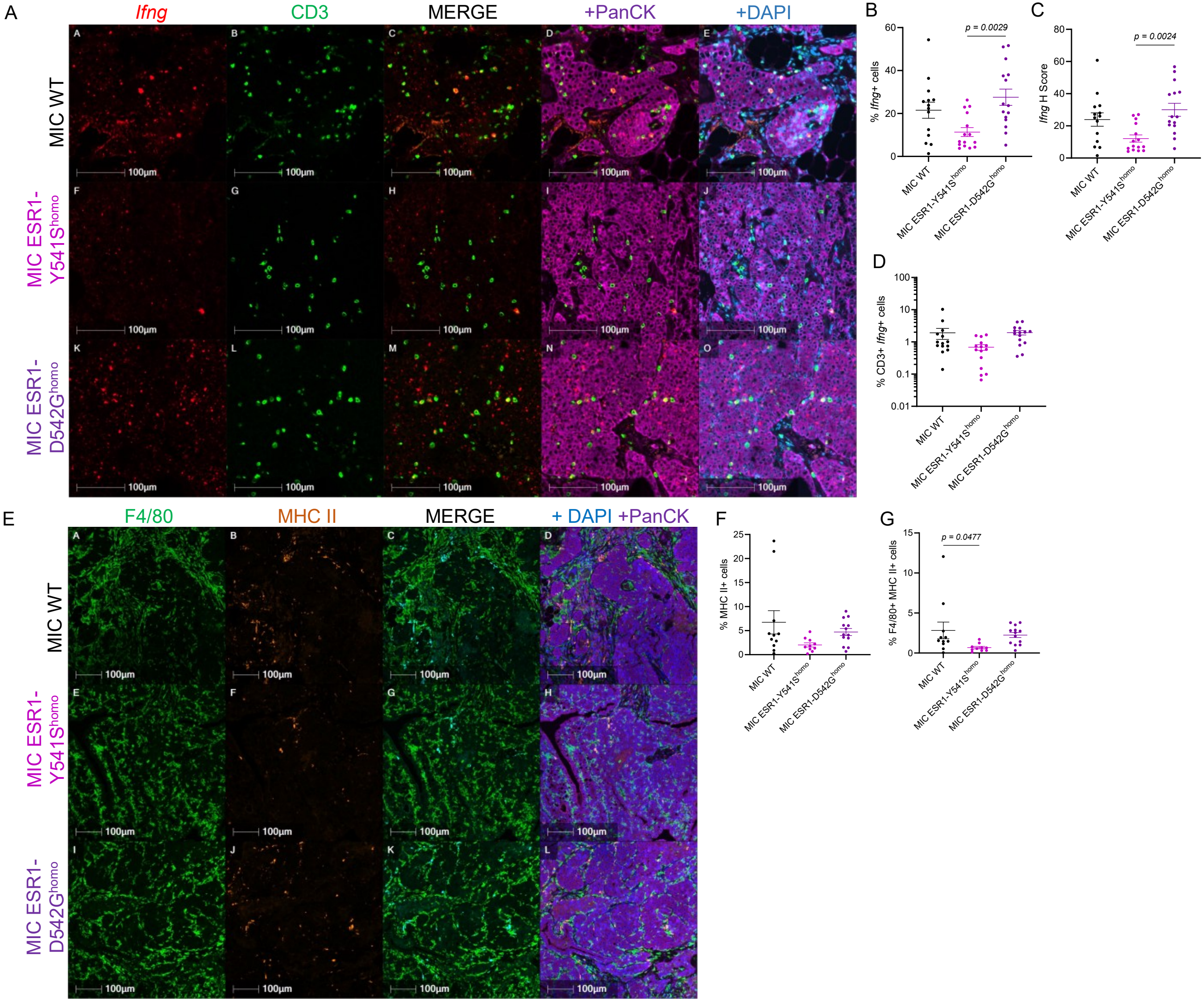

Supplemental Figure 5. MIC ESR1-Y541S^{homo} endpoint mammary tumors have decreased T cell and macrophage activities. A. RNA FISH for mouse *Ifng* and fluorescent IHC for CD3, PanCK, and DAPI on endpoint mammary tumors of MIC WT, MIC ESR1-Y541S^{homo}, and MIC ESR1-D542G^{homo} mice. B-D. Quantification of *Ifng*+ cells, *Ifng* H Score, and CD3+ *Ifng*+ cells, respectively. E. Fluorescent IHC for F4/80, MHC II, PanCK, and DAPI on endpoint mammary tumors of MIC WT, MIC ESR1-Y541S^{homo}, and MIC ESR1-D542G^{homo} mice. F-G. Quantification of MHC II+ cells and F4/80+ MHC II+ cells, respectively. Scale bars are as indicated on each image. Mean ± SEM for data calculated using one-way ANOVA with Tukey's multiple comparisons test.

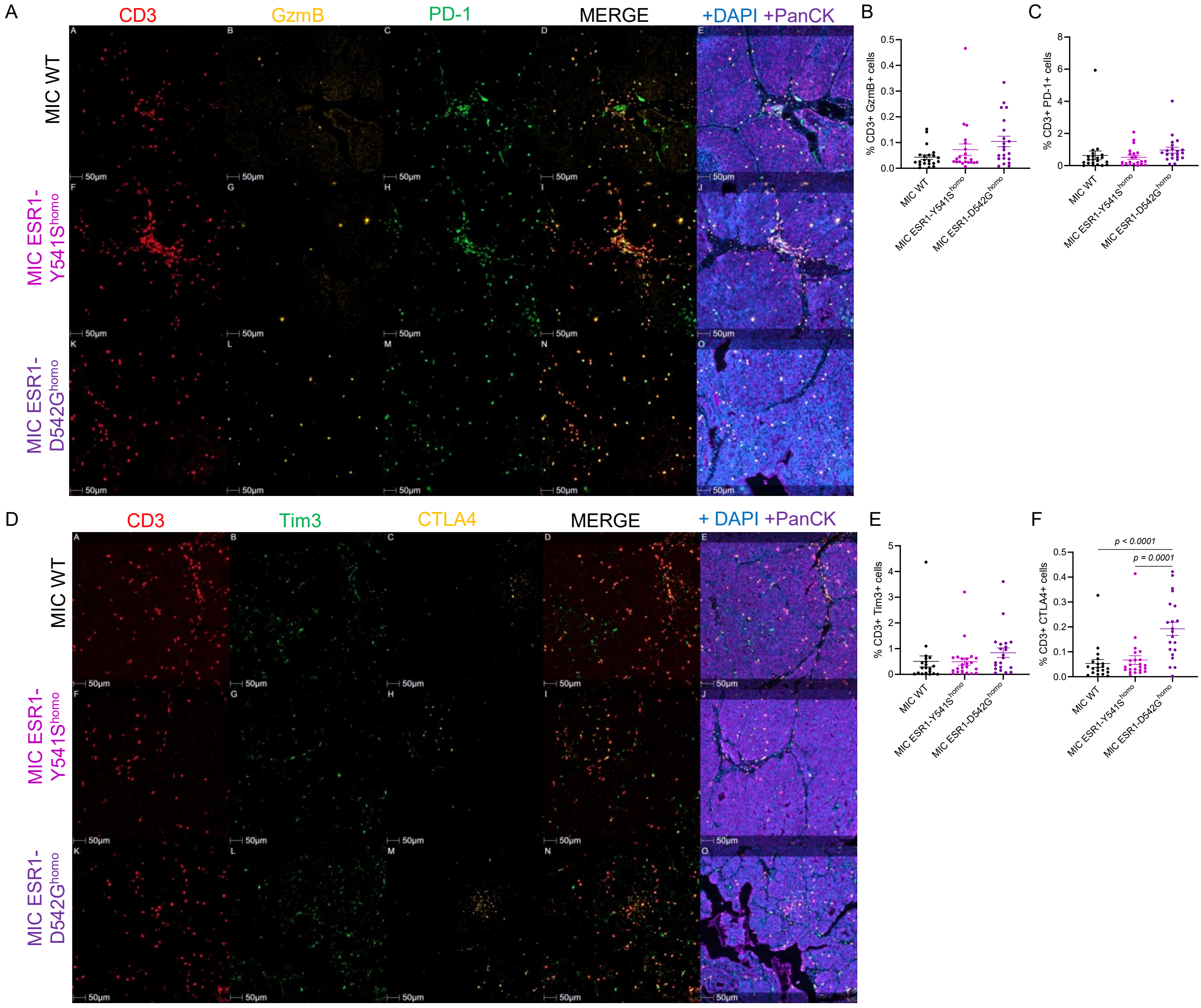

Supplemental Figure 6. Characterization of T cell activation and exhaustion in endpoint mammary tumors. A. Fluorescent IHC for CD3, granzyme B (GzmB), PD-1, PanCK, and DAPI on endpoint mammary tumors of MIC WT, MIC ESR1-Y541S^{homo}, and MIC ESR1-D542G^{homo} mice. B-C. Quantification of CD3+ GzmB+ cells and CD3+ PD-1+ cells, respectively. D. Fluorescent IHC for CD3, Tim3, CTLA4, PanCK, and DAPI on endpoint mammary tumors of MIC WT, MIC ESR1-Y541S^{homo}, and MIC ESR1-D542G^{homo} mice. E-F. Quantification of CD3+ Tim3+ cells and CD3+ CTLA4+ cells, respectively. Scale bars are as indicated on each image. Mean ± SEM for data calculated using one-way ANOVA with Tukey's multiple comparisons test.

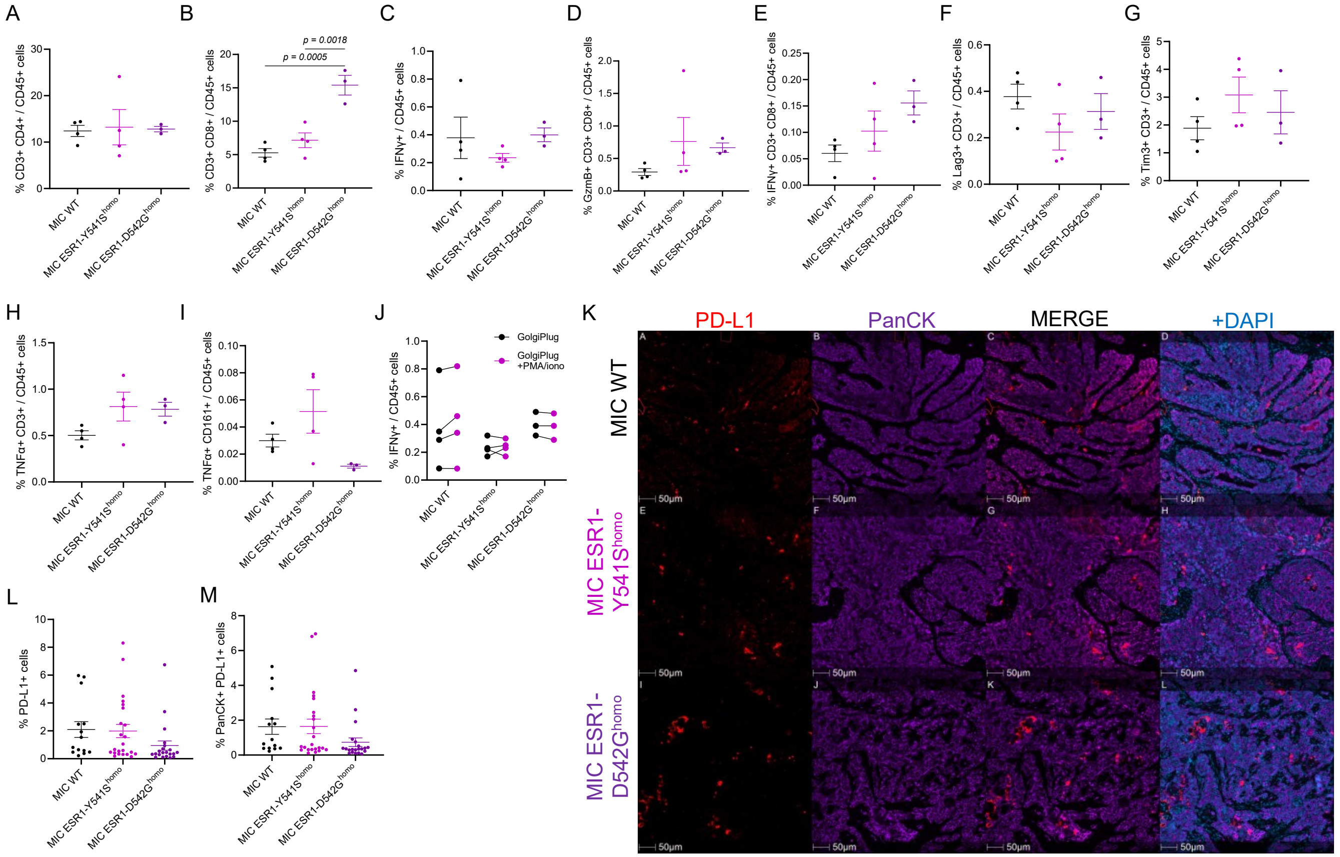

Supplemental Figure 7. Characterization of functional states of immune cells by flow cytometry in endpoint mammary tumors. A-I. Quantification of CD3+ CD4+ cells, CD3+ CD8+ cells, IFNγ+ cells, GzmB+ CD3+ CD8+ cells, IFNγ+ CD3+ CD8+ cells, Lag3+ CD3+ cells, Tim3+ CD3+ cells, TNFα+ CD3+ cells, and TNFα+ CD161+ cells out of total CD45+ cells by flow cytometry, respectively. J. IFNγ+ cells out of total CD45+ cells with or without PMA/ionomycin stimulation in endpoint mammary tumors of MIC WT, MIC ESR1-Y541S^{homo}, and MIC ESR1-D542G^{homo} mice by flow cytometry. K. Fluorescent IHC for PD-L1, PanCK, and DAPI on endpoint mammary tumors of MIC WT, MIC ESR1-Y541S^{homo}, and MIC ESR1-D542G^{homo} mice. L-M. Quantification of PD-L1+ cells and PanCK+ PD-L1+ cells, respectively. Scale bars are as indicated on each image. Mean ± SEM for data calculated using one-way ANOVA with Tukey's multiple comparisons test.

A

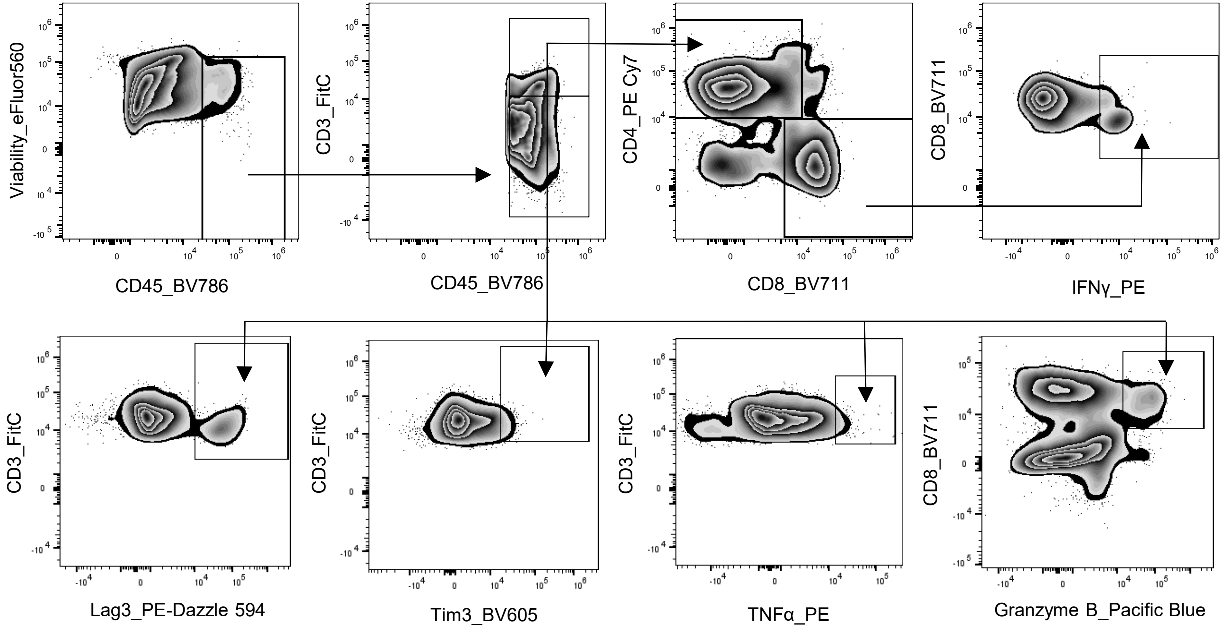

B

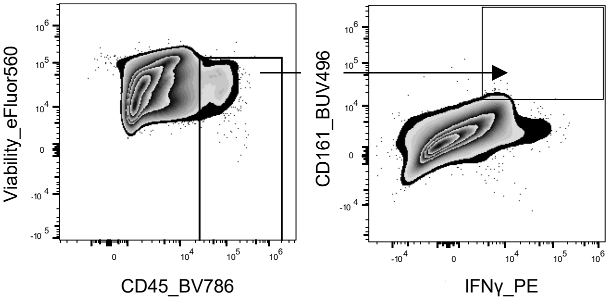

Supplemental Figure 8. Flow cytometry gating for immune cells and their functional markers on endpoint mammary tumors. A. Flow cytometry gating strategy for viable CD45+ immune cells, then CD3+ total T cells, then CD4+ T cells and CD8+ T cells. CD8+ T cells are gated for IFNγ+. CD3+ T cells are gated for Lag3+, Tim3+, TNFα+, and CD8+ granzyme B+ cells. B. Representative flow cytometry gating strategy for viable CD45+ immune cells then IFNγ+ CD161+ NK cells. A-B depict a representative sample for endpoint mammary tumors of MIC WT (n = 4), MIC ESR1-Y541S^{homo} (n = 4), and MIC ESR1-D542G^{homo} (n = 3) mice. Created on FlowJo.

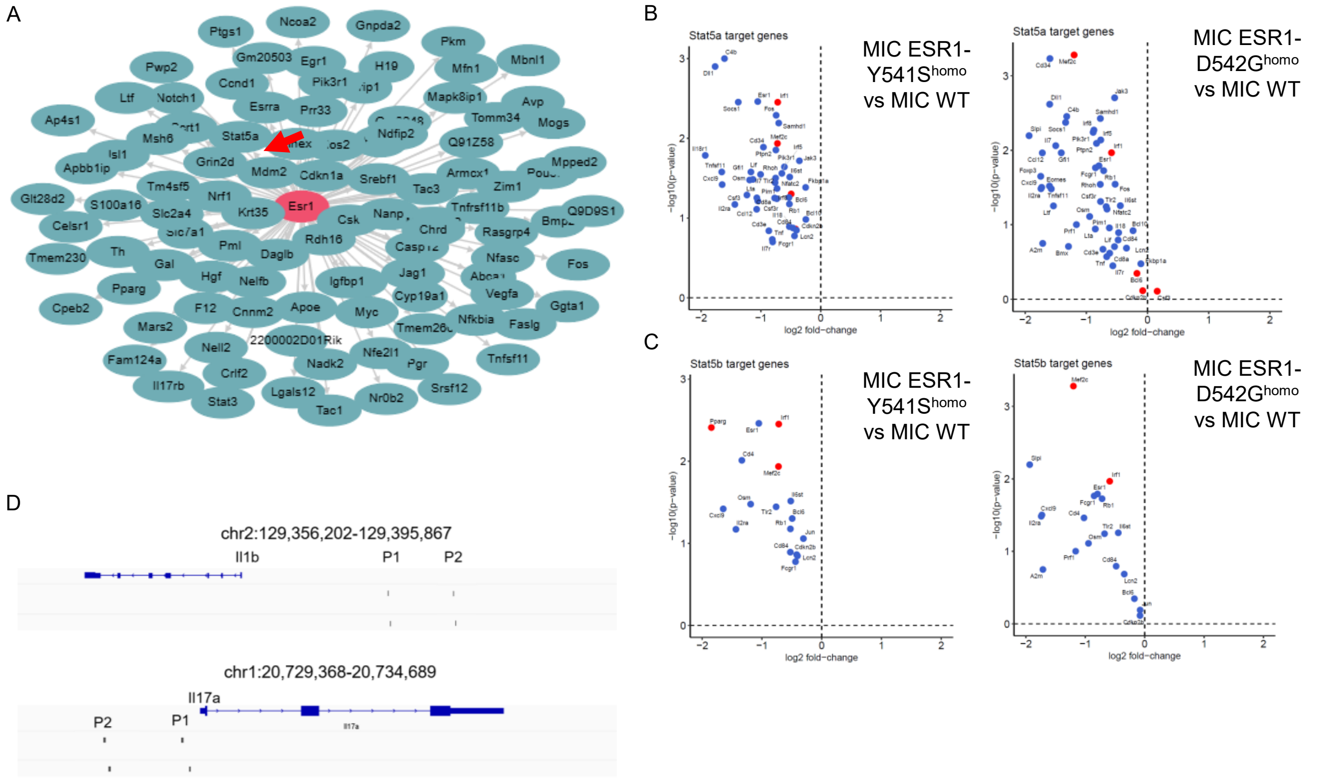

Supplemental Figure 9. Stat5 is a target of ERα and contributes to the decreased immune pathway activity in MIC ESR1^{mut} tumors. A. Transcription factor network for ERα (*Esr1*) indicating Stat5 as a transcription target of ERα using tflink.net. B-C. Volcano plot depicting Stat5a and Stat5b transcription factor-target genes contributing to the downregulated immune-related pathways in MIC ESR1-Y541S^{homo} or MIC ESR1-D542G^{homo} vs MIC WT tumors, respectively. Blue color denotes that the target genes are deactivated and concordant to the known direction of *ESR1^{mut}*-target interaction. Red color denotes that the target genes are deactivated but discordant to the known direction of *ESR1^{mut}*-target interaction. D. ChIP-qPCR primer positions for *Il1b* and *Il17a* promoters shown on IGV for Figure 8J.

A

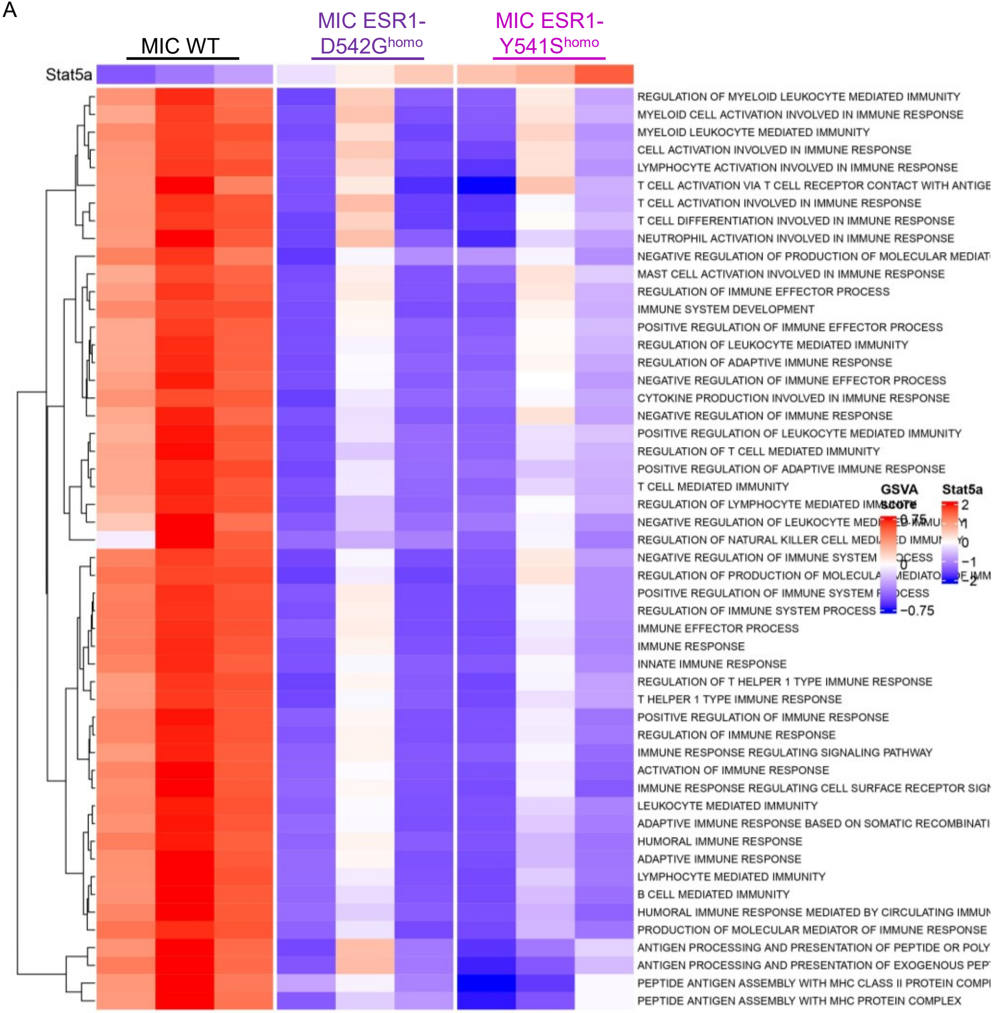

Supplemental Figure 10. High-Stat5a expression correlates with decreased immune pathway activity. A. Stat5a expression correlation map between MIC WT, MIC ESR1-Y541S^{homo}, and MIC ESR1-D542G^{homo} tumors and immune-related pathway activity from bulk RNA sequencing.

A

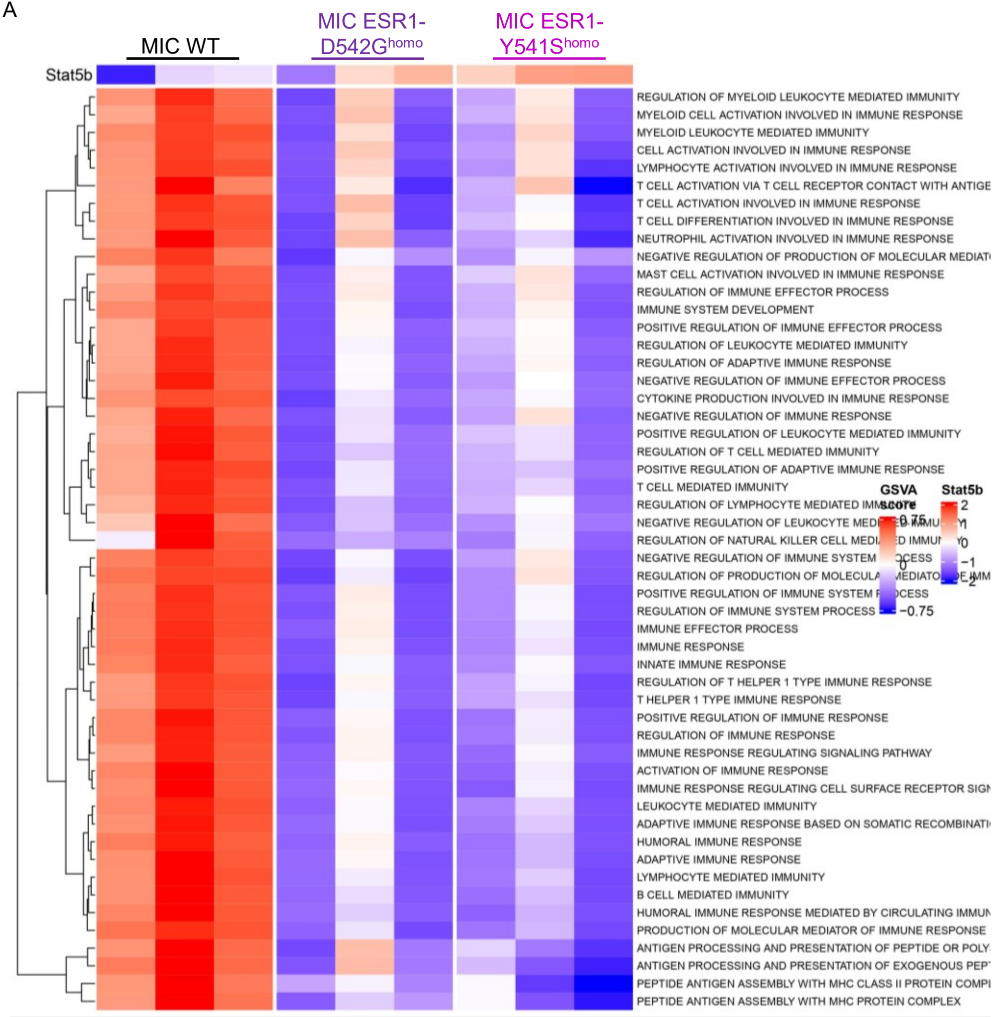

Supplemental Figure 11. High-Stat5b expression correlates with decreased immune pathway activity. A. Stat5b expression correlation map between MIC WT, MIC ESR1-Y541S^{homo}, and MIC ESR1-D542G^{homo} tumors and immune-related pathway activity from bulk RNA sequencing.

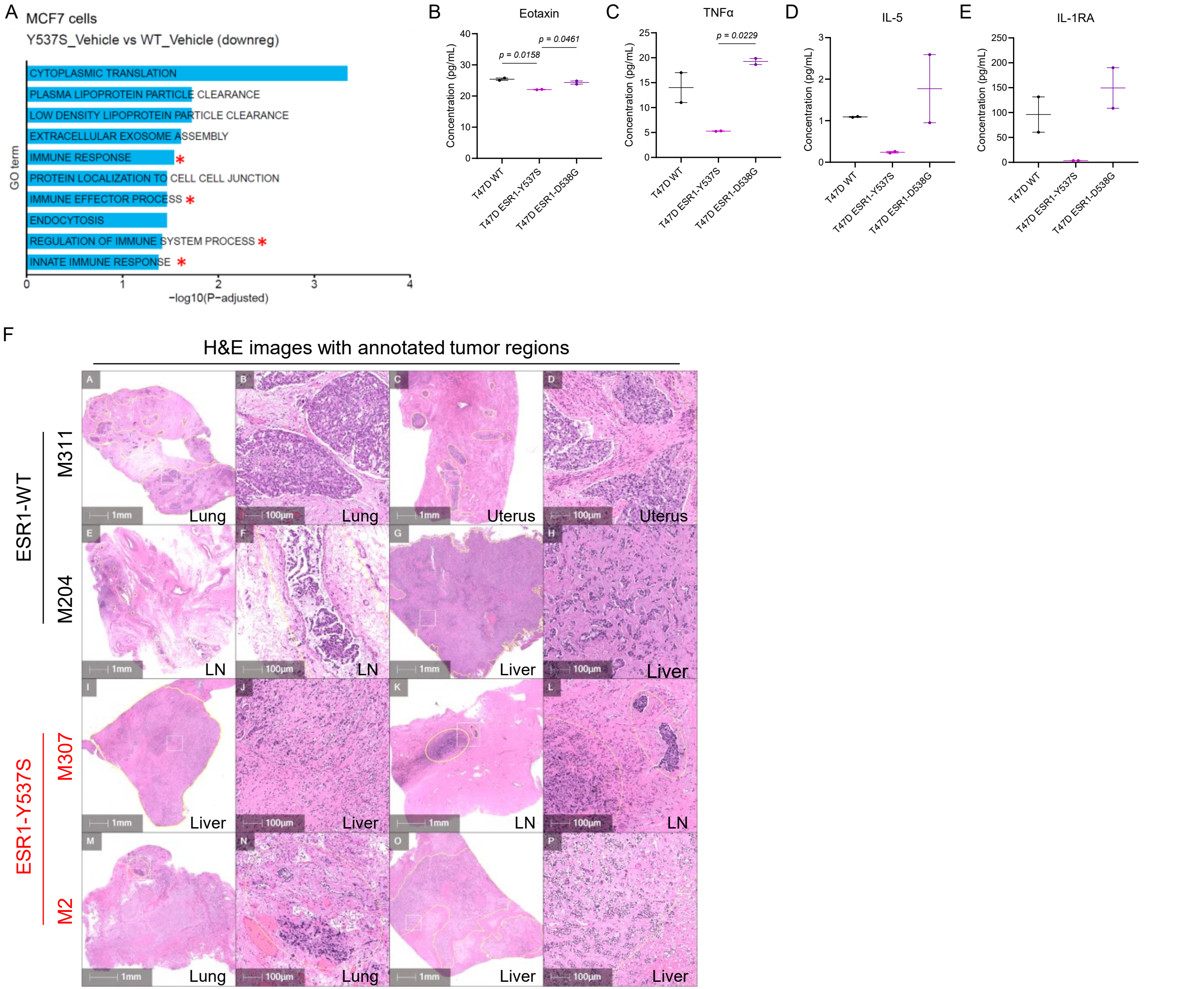

Supplemental Figure 12. *ESR1*^{Y537S} human breast cancer cell line and *ESR1*^{Y537S} metastatic breast cancer patient tumors analyses. A. Top Gene Ontology (GO) terms identified from downregulated DEG between MCF7 ESR1-WT and MCF7 ESR1-Y537S tumors from dataset GEO accession GSE266932, with stars delineating immune-related pathways. B-E. Quantification of observed concentrations of Eotaxin, TNFα, IL-5, and IL-1RA from cytokine multiplex assay on T47D WT, ESR1-Y537S, and ESR1-D538G human breast cancer cells, respectively. Each data point represents the mean of triplicate measurements. F. Representative H&E images of ESR1-WT and ESR1-Y537S patient metastases with annotations of pathological evaluation of tumor regions. Scale bars are as indicated on each image. Mean ± SEM for data calculated using one-way ANOVA with Tukey's multiple comparisons.

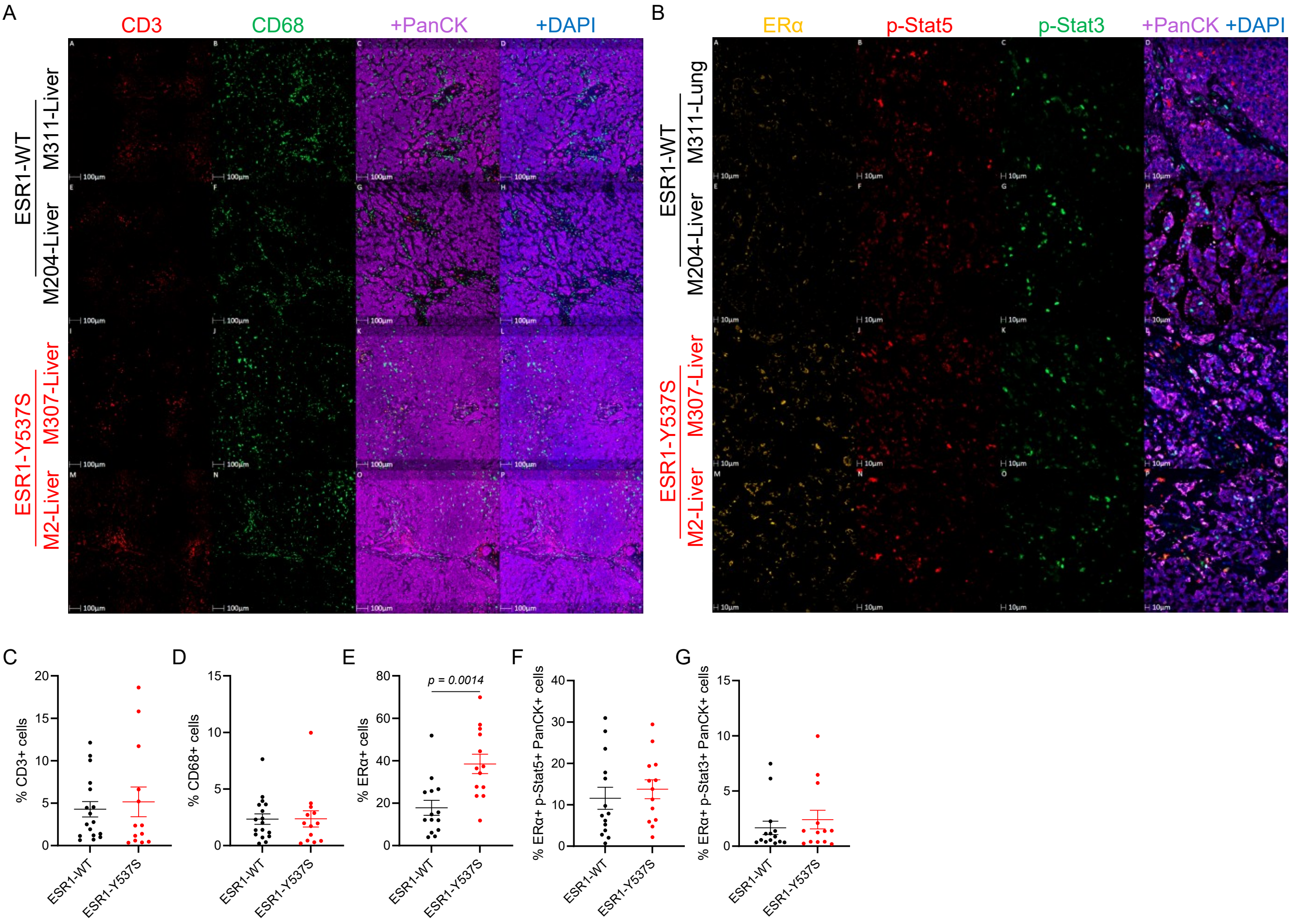

Supplemental Figure 13. *ESR1*^{Y537S} metastatic breast cancer patient tumors have comparable immune cell populations but elevated ERα levels. A. Fluorescent IHC for CD3, CD68, PanCK, and DAPI on ESR1-WT and ESR1-Y537S patient tumors. B. Fluorescent IHC for ERα, p-Stat5, p-Stat3, PanCK, and DAPI on ESR1-WT and ESR1-Y537S patient tumors. C-D. Quantification of CD3+ cells and CD68+ cells by ER mutation status, respectively. E-G. Quantification of ERα+ cells, ERα+ p-Stat5+ PanCK+ cells, and ERα+ p-Stat3+ PanCK+ cells by ER mutation status, respectively. Scale bars are as indicated on each image. Mean ± SEM for data calculated using two-tailed Student's t test.
