# Supplementary material for: Activating mutations in ESR1 contribute to an immunosuppressive breast tumor microenvironment by dampening cytokine secretion
Source: JCI Insight. 2026 Mar 9;11(5):e199927. doi: 10.1172/jci.insight.199927 (PMC13041671; doi:10.1172/jci.insight.199927)
Supplement: Unedited blot and gel images [file jciinsight-11-199927-s081.pdf]

Title:

Activating mutations in *ESR1* contribute to an immunosuppressive breast tumor microenvironment by dampening cytokine secretion

Authors:
Yu Gu [1, 2], Dongmei Zuo [1], Qixin Hu [1, 3], Virginie Sanguin-Gendreau [1], Alain Pacis [4], Marie-Christine Guiot [5], Alexander Chih-Chieh Chang [6, 7], Tarek Taifour [1, 8], Chen Ling [1], Adrian V Lee [6, 7], Steffi Oesterreich [6, 7], and William J. Muller [1, 2, 9]

Unedited blots

A

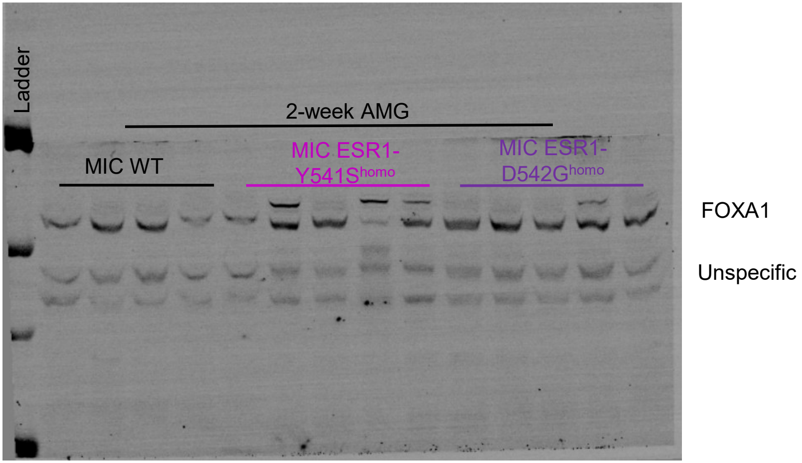

B

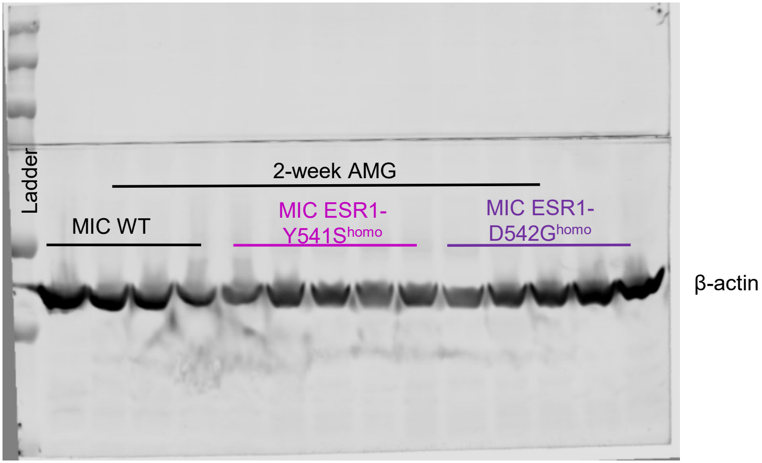

A-B. Full unedited blot/gel for Supplemental Figure 1B.

A

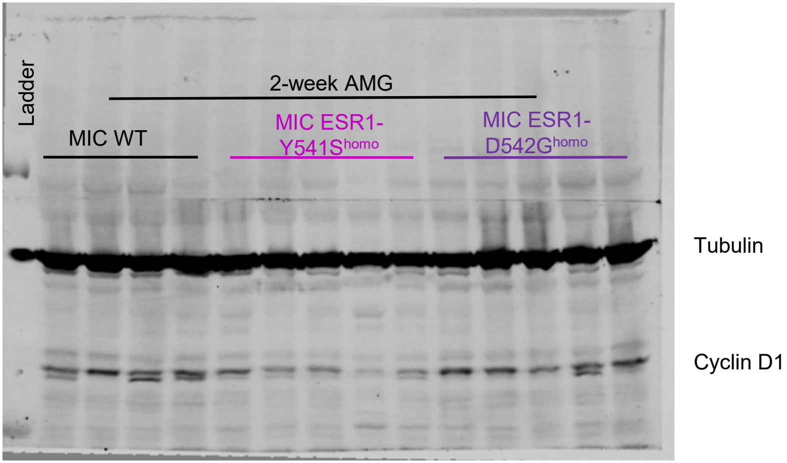

A. Full unedited blot/gel for Supplemental Figure 1B.

A

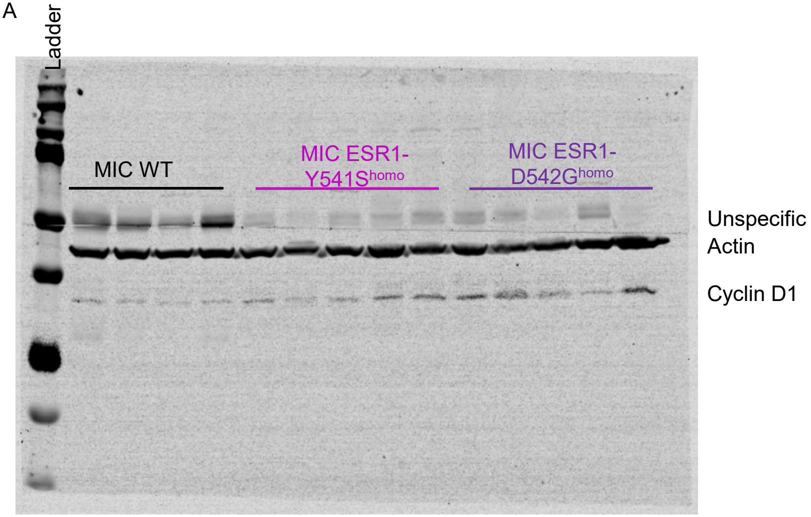

B

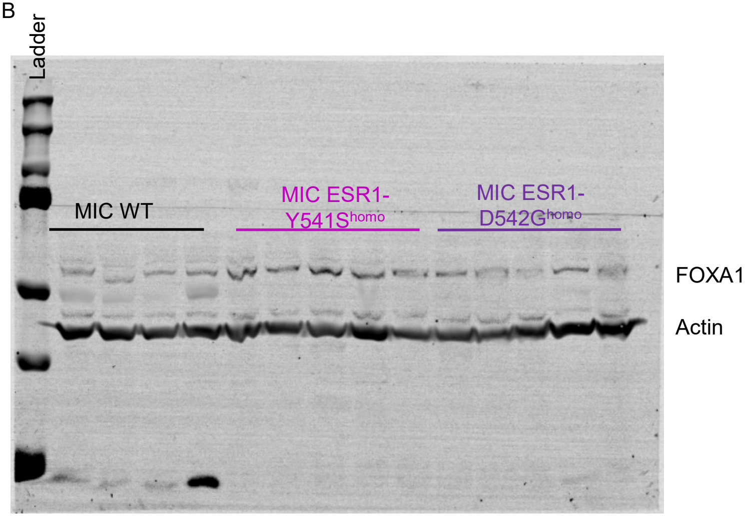

A-B. Full unedited blot/gel for Figure 2F.

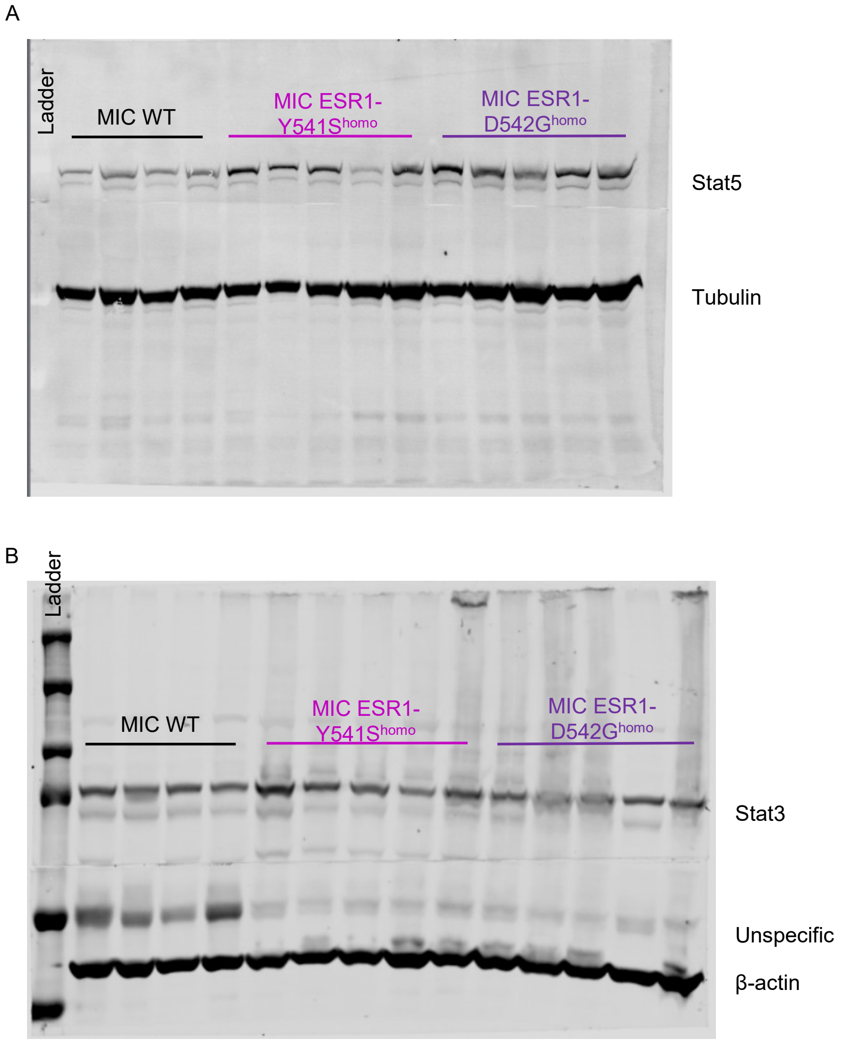

A-B. Full unedited blot/gel for Figure 8A.

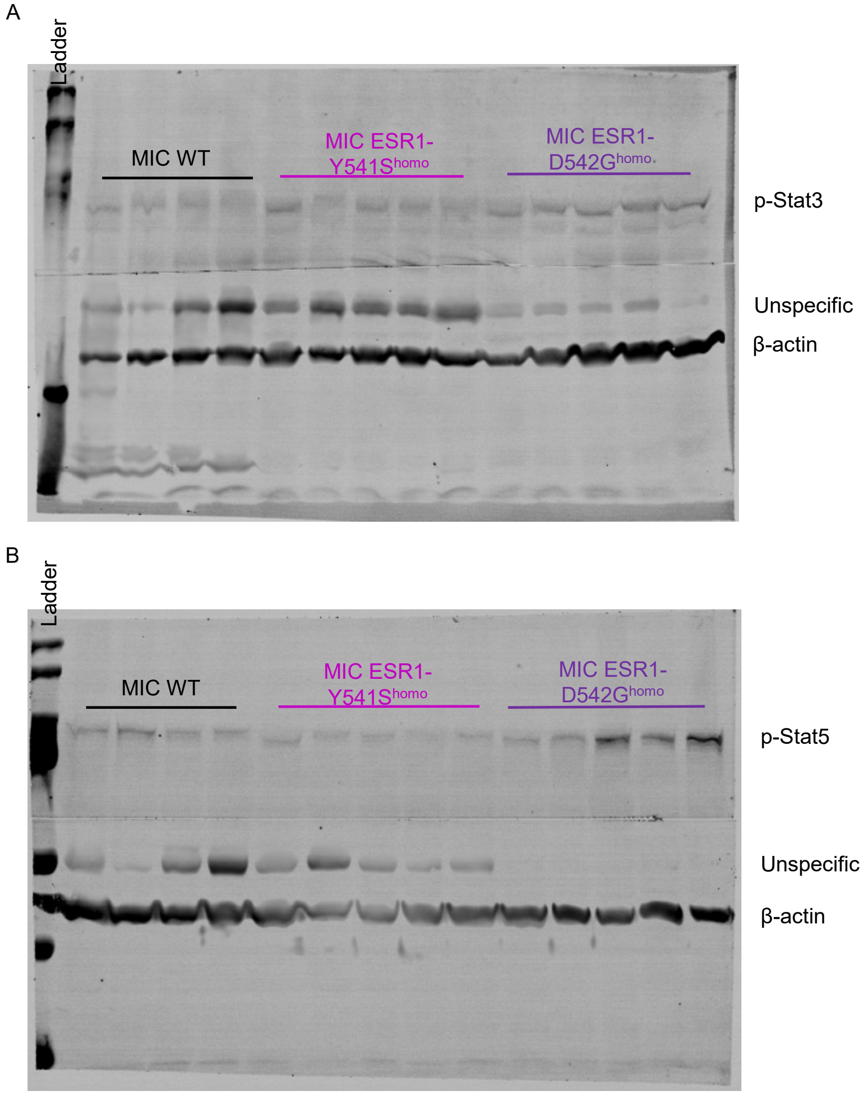

A-B. Full unedited blot/gel for Figure 8A.
